# The mechanism of Sanzi Yangqin decoction for asthma treatment based on network pharmacology and experimental verification

**DOI:** 10.1186/s12906-023-04272-6

**Published:** 2023-12-13

**Authors:** Yue Wu, Zhenhua Ni, Shiqiang Wang, Yipeng Sun, Xuming Luo, Xiongbiao Wang, Jinjin Liu

**Affiliations:** 1 Department of Respiratory Medicine, Putuo Hospital, Shanghai University of Traditional Chinese Medicine, Shanghai, 200062 China; 2 Central lab, Putuo Hospital, Shanghai University of Traditional Chinese Medicine, Shanghai, 200062 China

**Keywords:** SZYQD, Asthma, IL-6, AKT, Network pharmacology

## Abstract

**Background:**

Asthma is a chronic airway inflammatory disease characterized by airway inflammation, mucus hypersecretion, airway hyper-reactivity. Sanzi Yangqin Decoction (SZYQD) is widely prescribed for asthma treatment. Its anti-asthma activities have been reported in animal model, but the exact mechanism and targets of SZYQD in asthma treatment have not been fully elucidated.

**Methods:**

A network pharmacological approach was used to predict the active components, targets, and signalling pathways of SZYQD in asthma, including potential target prediction, protein‒protein interaction (PPI) network construction and analysis, and Gene Ont (GO) and Kyoto Encyclopedia of Genes and Genomes (KEGG) pathway analysis. The active ingredients were identified from the SZYQD, and were molecular docked according to the results of network pharmacology. A mouse model of asthma induced by ovalbumin (OVA) and lipopolysaccharide (LPS) was constructed to evaluate the therapeutic effect of SZYQD. Furthermore, the effects of SZYQD and its active ingredients were tested in vitro for regulating inflammation and MUC5AC expression (two main pathophysiologic abnormalities of asthma) in macrophages and airway epithelial cells by using Real-time PCR and western blotting.

**Results:**

A total of 28 active ingredients and 111 HUB genes were screened in the relevant databases, including three key ingredients (luteolin, β-carotene, and Sinapine) and nine core target genes (JUN, CTNNB1, IL10, TP53, AKT1, STAT3, TNF, IL6 and EGFR). KEGG and GO analysis indicated that the potential anti-asthmatic mechanisms of SZYQD were related to PI3K-Akt signalling pathway and response to lipopolysaccharide, etc. In the in vivo asthmatic model, our findings demonstrated that SZYQD exerted a protective effect against asthmatic mice induced by OVA and LPS through the inhibition of inflammation and mucus overproduction. Consistently, cell experiments showed that the SZYQD extract or the key active ingredients luteolin significantly decreased lipopolysaccharide (LPS)-induced IL-6 expression and activation of the NF-κB pathway in macrophages. In addition, SZYQD extract or luteolin inhibited activation of the AKT pathway and expression of MUC5AC induced by EGF in airway epithelial cells.

**Conclusion:**

The anti-asthmatic mechanism of SZYQD might be associated with inhibiting inflammation and airway mucus hypersecretion by regulating the NF-κB and AKT signalling pathways as predicted by network pharmacology, which provides more evidence for the application of SZYQD in asthma treatment.

**Supplementary Information:**

The online version contains supplementary material available at 10.1186/s12906-023-04272-6.

## Introduction

Asthma is a global disease, with more than 339 million cases according to the 2016 global disease burden study [1]. And it is expected to increase to 400 million by 2025. Patients with asthma and allergic diseases have a reduced quality of life. According to the World Health Organization, asthma causes 250,000 deaths each year [2]. Asthma is a chronic inflammatory disease of the airway that is composed of eosinophils, mast cells, neutrophils, and cellular components. The disease occurs under precipitating factors such as cold, the environment, and genetics. The gradual deterioration of the environment and air quality have also increased the incidence of asthma. The pathogenesis of asthma is complex and has not been clearly elucidated; airway inflammation, mucus hypersecretion, and airway remodelling are recognized as features of asthma [3]. The application of corticosteroids and long-acting inhaled β(2)-agonists for asthma treatment is effective, but a part of patients remain poorly controlled [4].

Clinically, traditional Chinese medicine has good curative effect in treating asthma [5]. The origins of SZYQD can be traced back to the renowned medical text “Han Shi Yi Tong” [6]. The composition of SZYQD comprises *Fructose Perillae*, *Semen Raphani*, and *Semen Sinapis Albae*. This prescription has gained widespread recognition and is frequently employed for the treatment of asthma [7–9]. For example, Wang et al. studied 60 cases of asthma patients and found that SZYQD combined with Compound Methoxyphenamine Hydrochloride Capsules had a better clinical effect [8]. Yang et al. found that SZYQD could improve symptoms and improve lung function in patients [9]. However, the mechanism of SZYQD in the treatment of asthma and its molecular targets have not been fully elucidated.

Network pharmacology refers to the selection of specific nodes, multiple targets, and multiple signalling pathways based on the theory of systems biology and biological system networks [10, 11]. It was especially useful to investigate the potential therapeutic mechanism and targets of TCM [12, 13]. Based on proteomics, systems biology and pharmacology, and the pathogenesis of disease, the drug-disease relationship was established, and the action points of drugs against diseases could be indicated and visualized. Therefore, based on network pharmacology, this study analyzed and screened the effective ingredients and targets of SZYQD, as well as the genes that intersect with diseases, and explored the mechanism and targets of SZYQD in treating asthma, which could provide new reference for the clinical application of SZYQD in asthma treatment.

## Materials and methods

### Active ingredients collection and target filtering

The TCMSP database (http://tcmspw.com/tcmsp.php) was used to query the components of SZYQD, including Perilla frutescens,Semen sinapis,Semen raphani to obtain the corresponding compounds. OB (oral bioavailability) > = 30% and DL (drug-like property) > = 0.18 were used as thresholds to screen the active compounds from the TCMSP. Furthermore, as supported by the available literature, additional compounds presented in SZYQD that potentially possessed therapeutic properties, but did not meet the aforementioned criteria of OB and DL, were included for comprehensive analysis [14–19]. In the TCMSP database, the target proteins of key drug pairs were obtained by selecting the “Related Targets” query and correcting the UniProt database (https://www.uniprot.org/). Humans were used as the research object, and a target protein library of key drugs was established.

### Corresponding target of asthma

We searched “asthma” through the Gene Cards database (http://www.genecards.org/) and OMIM database (https://omim.org/) to obtain related genes.

### Acquired common targets

According to the results obtained by the methods of “2.1” and “2.2”, the effectively screened common compound targets and disease targets were intersected to obtain an intersected target, the key targets of SZYQD for treating asthma, and a Venn diagram was drawn.

### Network construction

To demonstrate the multi compounds therapeutic features of SZYQD, PPI networks were built to analyse the target interactions. The interactions between proteins involved in SZYQD treatment of asthma were obtained from the PPI network. Human as the research species, set the minimum required interaction score to > 0.7, and other parameters remain unchanged by default. The results were imported into Cytoscape for network visualization processing, and a PPI role relationship diagram was constructed. Cytoscape was used to visually analyse the active components, targets, and disease genes, and a network diagram of active components–targets–disease was constructed. Through network topology analysis, targets with degrees of freedom, mediator numbers and centre numbers exceeding the average were regarded as the core targets.

### GO and KEGG analysis

Key targets were entered into the Metascape database for GO enrichment analysis, with the initial parameters defaulted. GO enrichment analysis was mainly divided into three biological aspects: biological processes, cellular components, and molecular functions. It was mainly applied to the functional classification of genes and target prediction in biology. The KEGG database (http://www.genome.ad.jp/kegg/) was established by the Kanehisa laboratory in 1995 and is commonly used for pathway analysis and annotation in network pharmacology [20–22]. KEGG enrichment analysis was used to explore the distribution of signalling pathways of key targets.

### SZYQD preparation

The Chinese herbal medicine was purchased from Shanghai Huayu Machinery Co., Ltd. in Shanghai, China. A set of SZYQD components is listed in Table 1. In brief, 2 groups of SZYQD components were boiled with 2 L of cold water, the extracted liquid was filtered and concentrated to 0.3 g/mL [23].

**Table 1:** Composition of SZYQD

Latin scientific name	Amount
Perilla frutescens	15 g
Semen sinapis	15 g
Semen raphani	15 g

### OVA + LPS-challenged mouse model of asthma and SZYQD treatment

Total of 26 Female BALB/c mice were purchased from Shanghai SLAC Laboratory Animal Co. (Shanghai, China), All experimental procedures complied with the International Standards of Animal Welfare and were approved by the Institute Animal Care and Use Committee of Shanghai University of Traditional Chinese Medicine (Shanghai, China). The animals were randomly divided into 4 groups(n = 6–7): Normal group (N), asthma (OVA + LPS) group (A), asthma + SZYQD low dose group (A + SL) and asthma + SZYQD high dose (A + SH). On days 0, 7 and 14, the A, A + SL and A + SH groups were sensitized via intraperitoneal (i.p.) injection of 100 µg of OVA (Sigma, USA) complexed with alum. On days 0, and 7, the A, A + SL and A + SH groups were sensitized via intraperitoneal (i.p.) injection of 1 µg of LPS (Sigma, USA). On days 15, 17, 19, 22, 23, and 24, the A, A + SL and A + SH group were intranasally administered 50 µL of OVA and 1 µg of LPS. Between days 15 and 24, the A + SL and A + SH group was intragastrically (i.g.) administered SZYQD (15 g/ kg and 45 g/kg) [23]. On day 25, mice were euthanized by cervical dislocation, lungs were harvested for further analysis.

### Haematoxylin and eosin (HE)

The lung tissue specimens were fixed in a formalin solution and subsequently underwent paraffin embedding. Slices were obtained from the embedded tissue and subjected to staining with HE solution. The resulting images were captured using a microscope (Olympus BX 43, Tokyo, Japan). The evaluation of inflammation was conducted based on the degree of infiltration of inflammatory cells surrounding the airway. A scoring system was employed to quantify the severity of inflammation observed, with the following criteria: no inflammation (0 points), less (1); more (2 points) circles (2), inflammatory cell ring (3), and large infiltration of inflammatory cells (4).

### Molecular docking verification

Molecular docking was performed using AutoDockTools 1.5.6. The core compound structure file (mol2) was downloaded from the TCMSP database and then converted to a 3D structure using ChemOffice software to minimize the structure energy. AutoDockTools 1.5.6 software was used to hydrogen bond the 3D structure and saved as a pdbqt file. Target proteins were obtained from the PDB and imported into PyMOL software for dehydration and ligand isolation. The prepared compounds and target files were opened in AutoDockTools 1.5.6 for the active site of each target protein using the Genetic Algorithm algorithm [24]. PyMOL was used to visualize and analyse the interactions involving the active compounds.

### SZYQD water extract preparation

Traditional Chinese herbs were purchased from Shanghai Hua Yu Machinery Co., Shanghai, China. The SZYQD water extract is mainly made by Perilla frutescens, Semen sinapis, Semen raphani. One set of SZYQD components is listed in Table 1.

Five set of mixed herbs with water were boiled. The filtrate was collected and centrifuged. The supernatant was harvested and concentrated. The concentrated sample was filtered through a 0.22 μm microporous membrane used for in vitro study. For water extraction, directly freeze dried to a concentrated decoction to obtain lyophilized powder 27.6 g. The extraction rate was 14.15%. The powder was dissolved in water for use as water extract of SZYQD (SZYQD-W) in vitro studies.

### SZYQD water extract detection by UHPLC-QE-MS

Weigh 20 mg of the SZYQD water extract and add 500 µL of extract (methanol: water = 4:1) with an internal standard concentration of 10 µg/mL. Then, add 500 µL of water with the same internal standard concentration. Vortex the mixture for 4 min, followed by sonication in an ice water bath for 1 h and incubation at 40℃ for 1 h. Centrifuge the samples at 12,000 rpm for 15 min. Carefully remove the supernatant through a 0.22 μm microporous filter. The LC-MS/MS analysis was performed using a UHPLC system (Vanquish, Thermo Fisher Scientific). The flow rate was set at 0.4 mL/min, and the sample injection volume was set at 5 µL. The mobile phase consisted of 0.1% formic acid in water (A) and 0.1% formic acid in acetonitrile (B). An Orbitrap Exploris 120 mass spectrometer coupled with Xcalibur software was used to acquire the MS and MS/MS data based on the IDA acquisition mode.

### Reagents

RPMI-1640 (Thermo Fisher Scientific, USA), DMEM (Thermo Fisher scientific, Waltham, MA, USA), BCA protein detection kit and cell lysis reagent (Beyotime Biotechnology, Jiangsu, China), antibodies to p-p65, p-AKT, p-EGFR (Cell Signaling Technology, Danvers, USA), Actin (Abcam, Cambridge, USA), Luteolin (MCE, Shanghai, China).

### Cell culture

The NCI-H292 human lung airway epithelial cells was provide by The Cell Bank of Type Culture Collection of Chinese Academy of Sciences (Shanghai, China) and MH-S mouse macrophages was provided by Shanghai Zhong Qiao Xin Zhou Biotechnology Company (Shanghai, China). The H292 airway epithelial cells were cultured in RPMI-1640 medium and murine alveolar macrophages (MH-S) were cultured in DMEM at 37 ℃ containing 5% CO_2_.

### Real-time quantitative polymerase chain reaction (PCR) (RT-qPCR)

Total RNA was extracted by an EZB kit and reverse transcribed into cDNA. qPCR primers are listed in Table 2. qPCR was carried out, and each sample was measured three times. The expression of related genes relative to β-actin was calculated by the 2-delta Ct method.

**Table 2:** Sequences of primers

Primers or probes	Sequence
mIL6-F	TCTATACCACTTCACAAGTCGGA
mIL6-R	GAATTGCCATTGCACAACTCTTT
MUC5AC-F	5- GCTTCCTGCTCCGAGATGT−3
MUC5AC-R	5-AAGACGCAGCCCTCATAGAA−3
m-actin-F	GGCTGTATTCCCCTCCATCG
m-actin-R	CCAGTTGGTAACAATGCCATGT
hβ-actin-F	5-CCAACCGCGAGAAGATGA−3
hβ-actin-R	5-CCAGAGGCGTACAGGGATAG−3

### Enzyme-linked immunosorbent assay (ELISA)

M-HS cells were treated with LPS for 24 h, and IL-6 in cell culture supernatants was determined using a mouse IL-6 ELISA kit (ABclone) according to the manufacturer’s instructions.

### Western blot

Cells were lysed with RIPA buffer. The proteins were separated by sodium dodecyl sulfate‒polyacrylamide gel electrophoresis (SDS-PAGE) and transferred to polyvinylidene fluoride membranes (PVDF). The membrane was blocked with 5% milk at room temperature for 2 h. Membranes were cropped along with the molecular weight marker to separate the target regions, and the primary antibodies p-P65, p-AKT, and p-EGFR were added to incubate the membrane overnight at 4 ℃, respectively. The membrane was then washed with TBST three times followed by incubation with anti-rabbit IgG horseradish peroxidase secondary antibody (Cell Signaling Technology, USA) for 2 h at room temperature. Finally, immunoreactive bands were visualized with ECL reagent.

### Statistics

SPSS 22.0 software and GraphPad Prism 8.0 were used to analyse the data. The results are expressed in the form of average standard deviation. T test, one-way analysis of variance, or two-way analysis of variance were used. A statistically significant difference was defined as p < 0.05.

## Results

### Active ingredients and targets of SZYQD

**Fig. 1:**
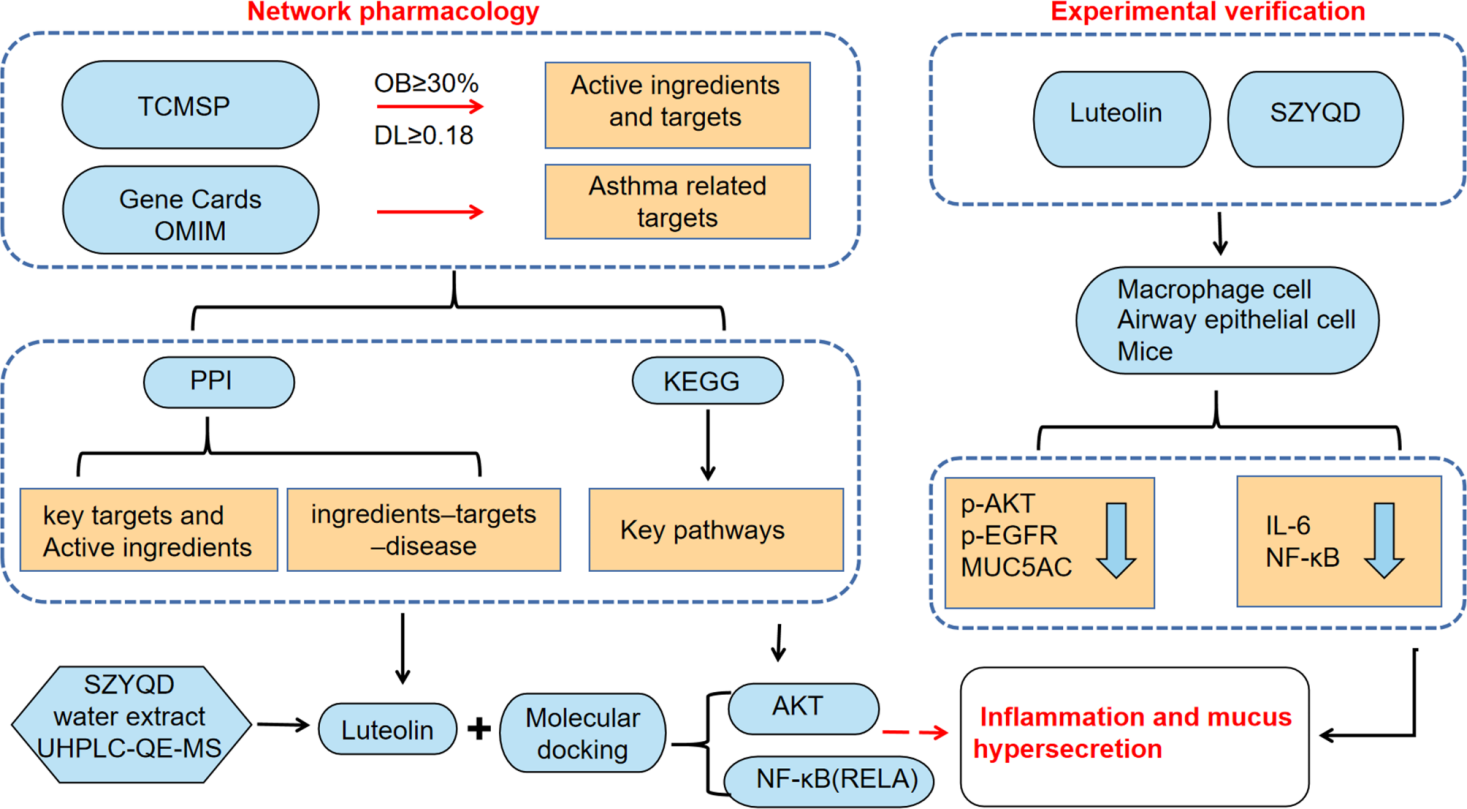
The basic process of network pharmacological research and experimental validation of SZYQD treatment for asthma, including database preparation, network construction, GO and KEGG pathway analysis, molecular docking validation, and Validation in cell and animal experiments

The flowchart of the study design is illustrated in Fig. 1. To screen theactive ingredients, OB (oral bioavailability) > = 30% and DL (drug-like property) > = 0.18 were set as thresholds in the TCMSP database. Additionally, in accordance with relevant reports, other active ingredients known to possess therapeutic effects in SZYQD were also included in the analysis. By applying these criteria and considering the broader spectrum of active constituents, a comprehensive compilation of 28 active ingredients was obtained (Table 3). According to the number of targets, luteolin, Sinapine, and β-sitosterol are the major pharmacological components in SZYQD.

**Table 3:** Active ingredients in SZYQD

Mol ID	Molecule Name	OB(%)	DL	Target
MOL000006	Luteolin	36.16	0.25	57
MOL001439	Arachidonic acid	45.57	0.2	38
MOL000358	Beta-sitosterol	36.91	0.75	38
MOL000449	Stigmasterol	43.83	0.76	31
MOL002773	Beta-carotene	37.18	0.58	22
MOL001697	Sinoacutine	63.39	0.53	18
MOL012893	(E)-(4-methylbenzylidene)-(4-phenyltriazol-1-yl)amine	57.87	0.19	5
MOL000953	CLR	37.87	0.68	4
MOL000359	Sitosterol	36.91	0.75	3
MOL004355	Spinasterol	42.98	0.76	3
MOL007449	24-methylidenelophenol	44.19	0.75	3
MOL009681	Obtusifoliol	42.55	0.76	3
MOL005030	Gondoic acid	30.7	0.2	2
MOL010672	icosa-8,11,14-trienoic acid methyl ester	44.81	0.23	1
MOL012888	Citrostadienol	43.28	0.79	1
MOL005043	campest-5-en-3beta-ol	37.58	0.71	1
MOL005481	2,6,10,14,18-pentamethylicosa-2,6,10,14,18-pentaene	33.4	0.24	1
MOL003975	icosa-11,14,17-trienoic acid methyl ester	44.81	0.23	0
MOL012891	(2E,4E,6E)-icosa-2,4,6-trienoic acid	41.64	0.2	0
MOL009653	Cycloeucalenol	39.73	0.79	0
MOL010690	Uniflex BYO	30.13	0.25	0
MOL013037	2-(2-phenylethyl)-6-[[(5 S,6R,7R,8 S)-5,6,7-trihydroxy-4-keto-2-(2-phenylethyl)-5,6,7,8-tetrahydrochromen-8-yl]oxy]chromone	31.31	0.61	0
MOL011865	Rosmarinic acid	1.38	0.35	30
MOL001254	Perillaldehyde	39	0.03	7
MOL006232	Perilla ketone	1.18	0.03	2
MOL002152	Sinapic acid	64.15	0.08	9
MOL010681	Sinapine thiocyanate	1.21	0.18	5
MOL010680	Sinapine	1.21	0.18	7

### Disease target and network construction

**Fig. 2:**
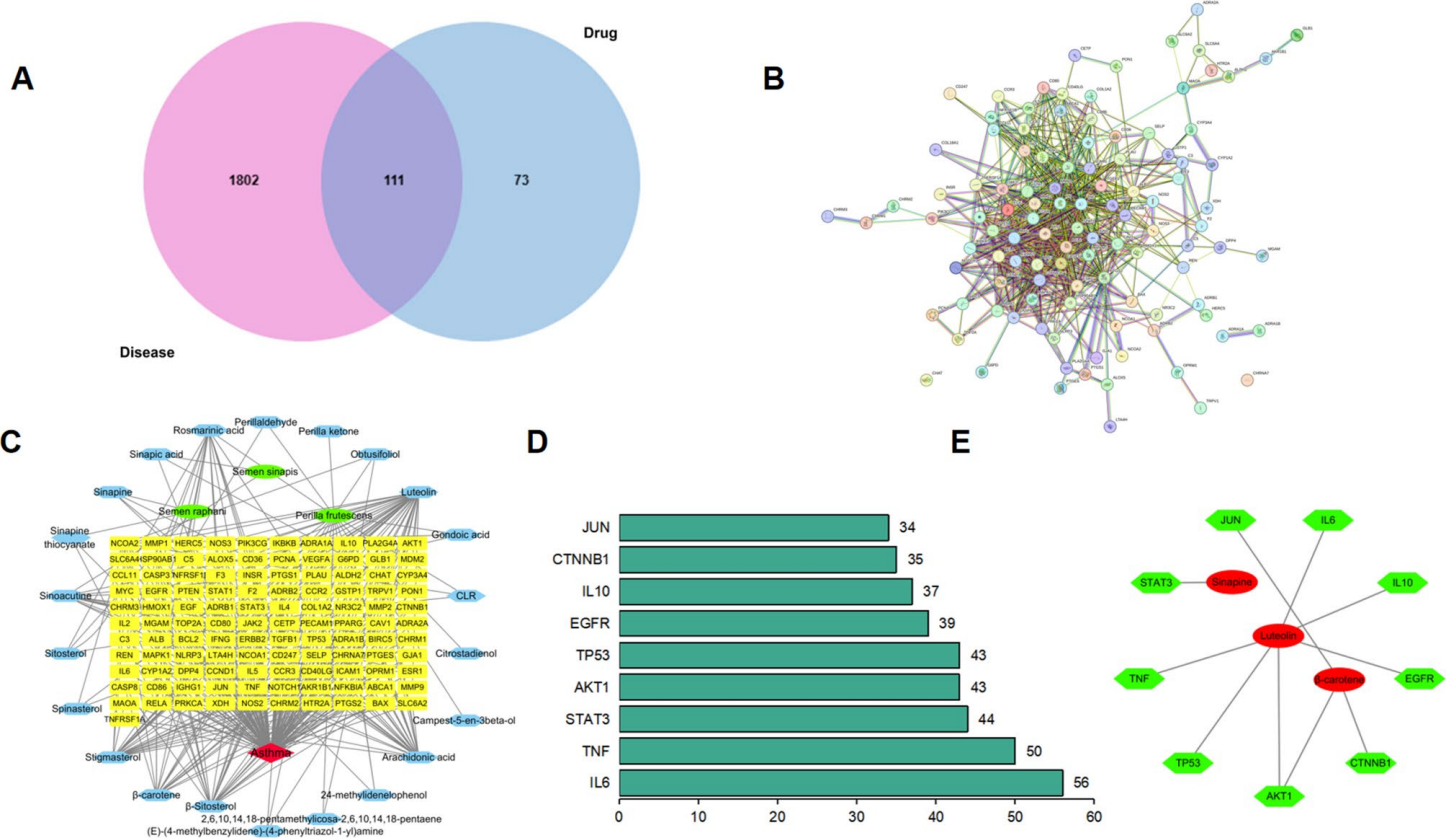
Disease target and network construction. (**A**) Network diagram of active components and targets of SZYQD. (**B**) PPI network of key targets. (**C**) SZYQD medicine-active ingredients-target-disease network. (**D**) Bar graph of the top 9 core targets. (**E**) Core targets and core target-compound

A total of 7547 genes were obtained by retrieving the Gene Cards and OMIM databases, including 1913 genes with relevance scores greater than 0.804, which were integrated and deduplicated with drug targets, and 111 HUB target proteins were finally obtained (Fig. 2A). The STRING database was used to analyse 111 key target proteins. The minimum required interaction score value was set to > 0.7, and then a protein‒protein interaction network analysis diagram (PPI) was constructed (Fig. 2B). The network included 111 nodes, 799 sides, and 34.3 average node degrees in the network. The results were imported into Cytoscape 3.7.1 to draw a visual model (Fig. 2C). The targets at the centre of this network were JUN, CTNNB1, IL10, TP53, AKT1, STAT3, TNF, IL6 and EGFR (Fig. 2D). This indicated that the above central target proteins might be closely related to the effect of asthma treatment with SZYQD. Further analysis showed that the above 9 core targets come from three key active compounds: luteolin, β-carotene, and Sinapine (Table 4; Fig. 2E).

**Table 4:** Core target-compound

Core target	Compound
AKT1	Luteolin,beta-carotene
IL6	luteolin
JUN	beta-carotene
IL10	luteolin
TNF	luteolin
TP53	luteolin
CTNNB1	beta-carotene
EGFR	arachidonic acid
STAT3	Sinapine

### GO and KEGG analysis

**Fig. 3:**
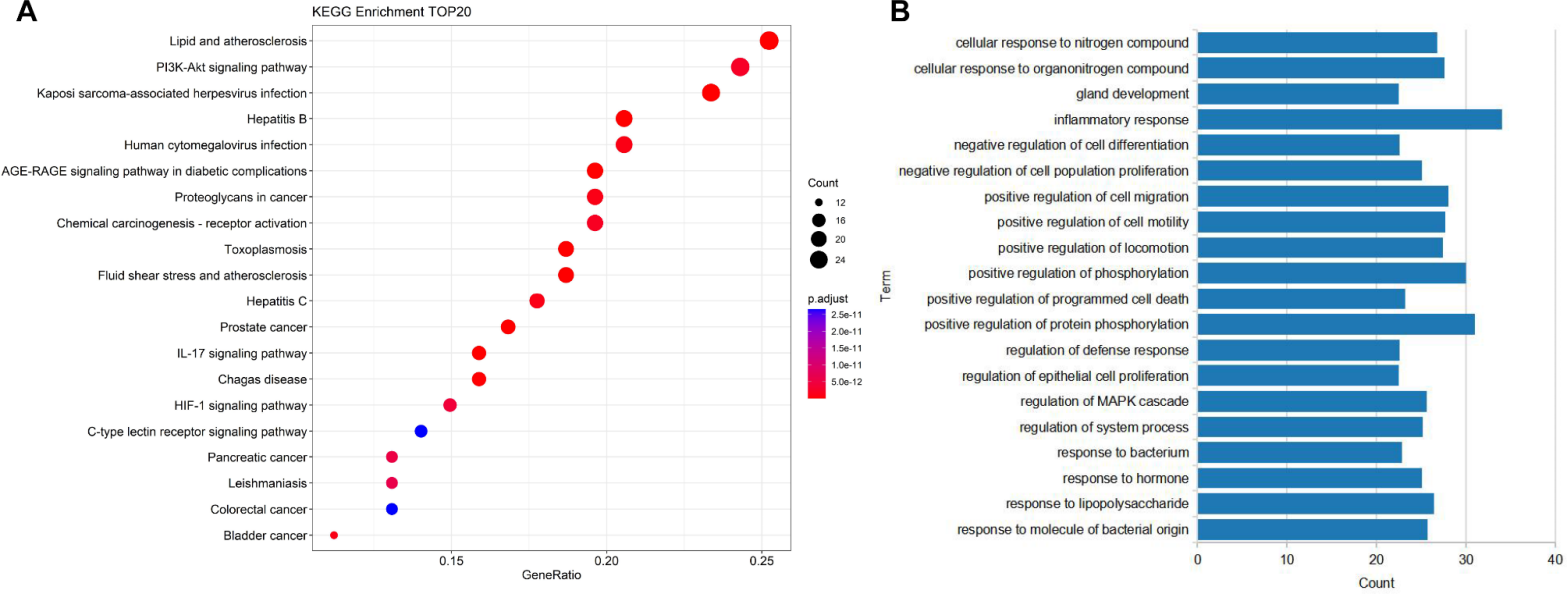
The bubble chart of the top 20 pathways based on KEGG enrichment. Analysis and GO enrichment analysis

To explore the functional distribution of SZYQD in asthma, 111 key targets were input into the Metascape database for GO enrichment analysis and KEGG pathway analysis. After analysis of the KEGG database, 5107 biological processes, 408 cellular components, and 811 molecular functions were found to be associated with the anti-asthmatic effect of SZYQD, involving 273 pathways. KEGG enrichment analysis was performed on 111 key targets, and 463 results were obtained, among which the first 20 significant items were selected (Fig. 3). The related pathways involved include Lipid and atherosclerosis signalling pathway, PI3K-Akt signalling pathway, Kaposi sarcoma-associated herpesvirus infection signalling pathway, antiviral infection (hepatitis B) signalling pathway, Human cytomegalovirus infection signalling pathway, AGE-RAGE signalling pathway, Cancer pathway. KEGG results show that the PI3K-Akt signalling pathway is one of the important target pathways in the treatment of asthma with SZYQD.

One hundred and one key targets were input into the Metascape database for GO enrichment analysis, and the threshold value P < 0.01 was set. The first 20 significant items were selected and plotted, and the results showed that the biological process involves positive regulation of protein phosphorylation, response to lipopolysaccharide, inflammatory response, and so on. Cellular components include asymmetric synapse, cytoplasmic side of membrane, and clathrin-coated vesicle, and molecular functions include metalloendopeptidase activity, electron transfer activity, and chromatin DNA binding and so on.

### Protective effects of SZYQD in an OVA + LPS-induced asthma mouse model

**Fig. 4:**
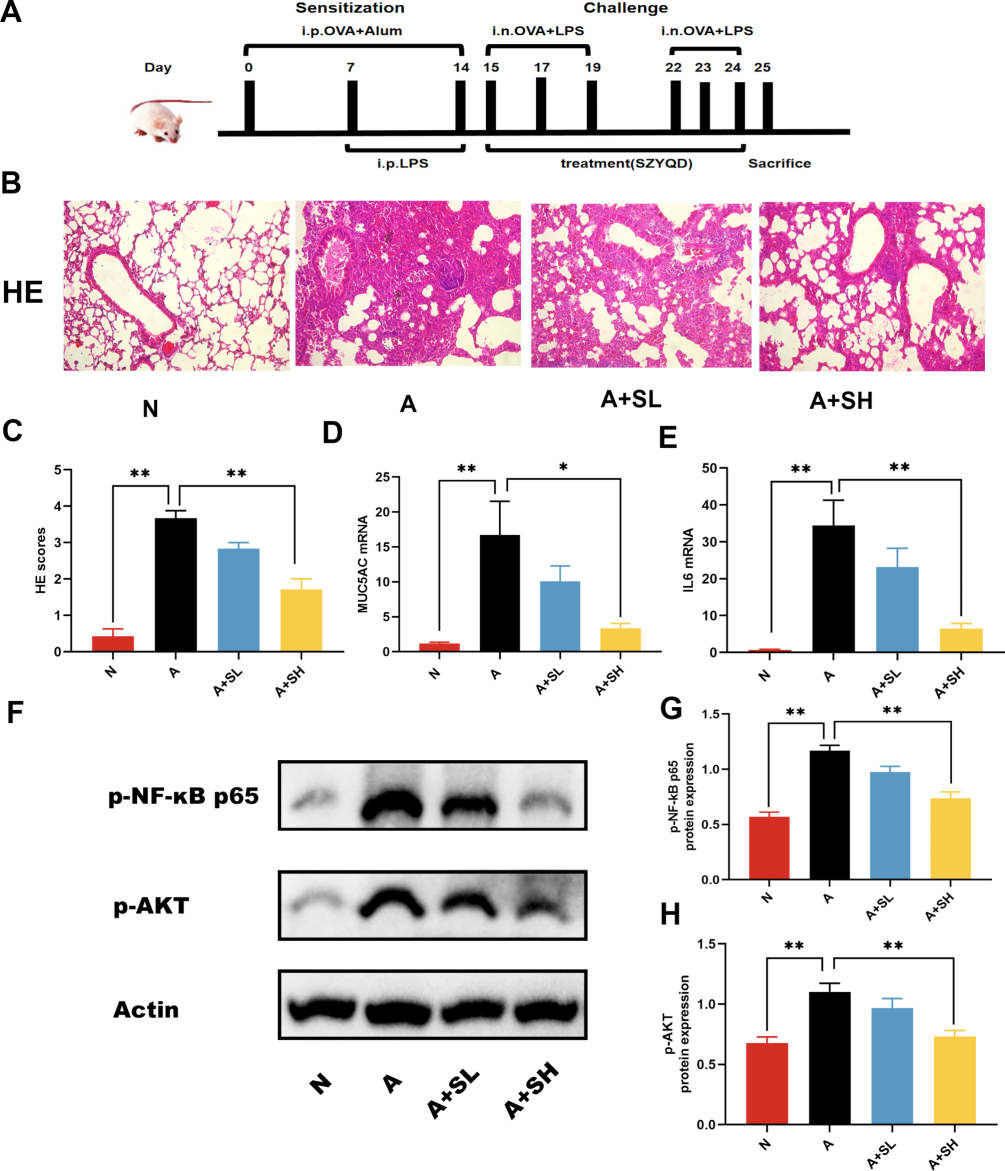
Protective effects of SZYQD in the OVA + LPS asthmatic model. (**A**) the procedure of OVA + LPS asthmatic model. (**B**) HE staining of lung tissue in different groups. Normal (N), asthma model (**A**), SZYQD low-dose treated (A + SL) group and SZYQD high-dose treated (A + SH) groups. Magnification: 200X. (**C**) HE scores in different groups. (**D-E**) mRNA expression of MUC5AC and IL6 in mouse lung was detected with RT-qPCR. (**F**) The expression levels of p-AKT and p-p65 protein levels were measured by western blot. (**G-H**) Quantification of p-p65 and p-AKT. Data mean ± S, ** P < 0.01,* P < 0.05, n = 6–7

GO analysis revealed that the protective effects of SZYQD might be attributed to its ability to modulate the response to lipopolysaccharide (LPS). In light of this finding, an OVA + LPS asthmatic model was established to further investigate the therapeutic potential of SZYQD (Fig. 4A). In this mouse model of asthma, the asthmatic group exhibited a notable increase in inflammatory cell infiltration surrounding the airways compared to the normal group. However, following treatment with a high dose of SZYQD, a significant reduction in infiltrated inflammatory cells was observed in the A + SH groups. (Fig. 4B-C). Additionally, we observed that SZYQD treatment effectively suppressed the overexpression of MUC5AC (Fig. 4D), which is considered a key pathophysiological abnormality in asthma. These findings provided further evidence of the protective role of SZYQD in the treatment of asthma, as it effectively regulates both inflammation and excessive mucus production.

In order to gain deeper insights into the underlying mechanisms of SZYQD in its anti-asthmatic effects, we selected IL-6, AKT, and RELA (p65, a subunit of NF-κB) for further validation. These specific targets were chosen based on the results obtained from network pharmacology analysis and their association with inflammation and mucus overproduction. We observed a significant increase in the relative levels of IL-6 mRNA in group A compared to group N, indicating an upregulation of IL-6 in asthma. However, following treatment with high doses of SZYQD, the expression of IL-6 was downregulated (Fig. 4E). Furthermore, the phosphorylation levels of proteins p-P65 and p-AKT were found to be significantly elevated in the asthma group. However, in the high-dose SZYQD group, the content of p-P65 and p-AKT was significantly lower compared to the asthma group (Fig. 4F-H). These findings suggested that SZYQD might exert its effects through the modulation of the AKT and NF-κB pathways. By targeting these specific pathways, SZYQD demonstrated its potential in ameliorating inflammation and excessive mucus production associated with asthma.

### SZYQD water extract inhibited LPS-induced IL-6 production and NF-κB activation in M-HS cells

**Fig. 5:**
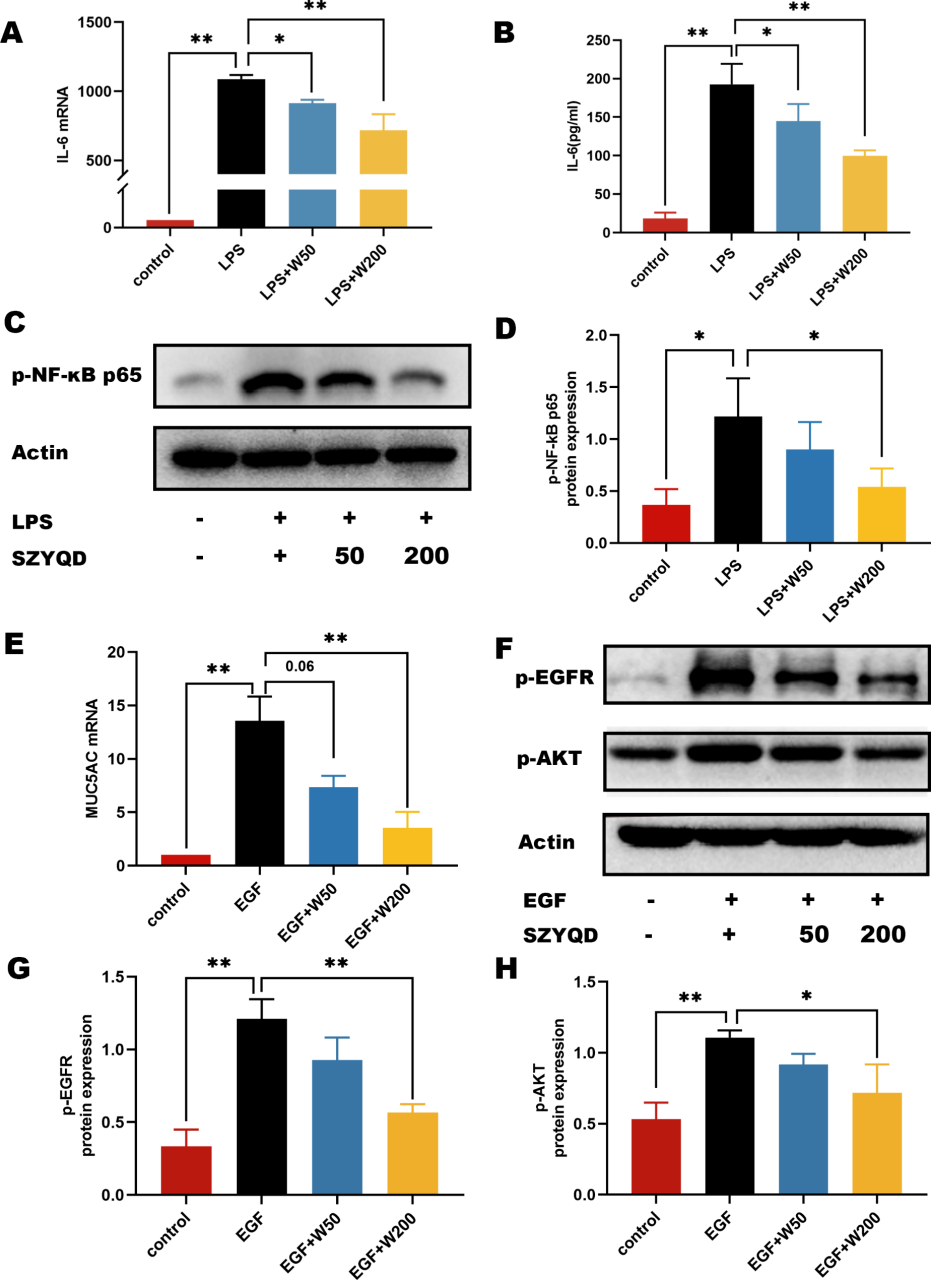
Effects of SZYQD water extract on LPS induced IL-6 expression in M-HS cells EGF induced activation of AKT signalling in H292 cells. (**A**) M-HS cells were exposed to LPS (1 µg/mL) and SZYQD water extract (50 µg/mL and 200 µg/mL) for 24 h. The expression levels of IL-6 were examined by qPCR. (**B**) Supernatants of M-HS cells were collected and IL-6 secretion levels were measured by ELISA. (**C**) The expression levels of p-p65 were measured by western blot after LPS (1 µg/mL) and SZYQD extract (50 µg/mL and 200 µg/mL) treatment for 1 h. (**D**) Quantification of p-p65. (**E**) H292 cells were exposed to EGF (50 ng/mL) and SZYQD water extract (50 µg/mL and 200 µg/mL) for 24 h. The expression levels of MUC5AC were examined by qPCR. (**F**) The expression levels of p-AKT and p-EGFR were measured by western blot after EGF (50 ng/mL) and SZYQD water extract (50 µg/mL and 200 µg/mL) treatment for 2 h. (**G-H**) Quantification of p-EGFR and p-AKT. The data were expressed as mean ± SD (n = 3) of each group. *p < 0.05, **p < 0.01

To investigate whether SZYQD exerted its effects through the aforementioned signaling pathways, in vitro experiments were conducted. We used LPS to stimulate IL-6 production and NF-κB activation in M-HS cells. Our results showed that compared with the control group, the expression of IL-6 in the LPS stimulation group was significantly increased, and the level of IL-6 decreased in a dose-dependent manner after intervention with different concentrations of SZYQD water extract (Fig. 5A-B). Furthermore, we found that the increased level of p-p65 was inhibited by SZYQD water extract treatment. These results demonstrated that SZYQD may have an anti-inflammatory effect by inhibiting LPS-induced IL-6 production and NF-κB activation in macrophages (Fig. 5C-D).

### SZYQD water extract inhibits the activation of the AKT signalling pathway and MUC5AC production induced by EGF in airway epithelial cells

Our in vivo results showed that the AKT pathway was related to the anti-asthmatic effect of SZYQD. It has been reported that the secretion of EGF increases in asthma and EGF can induce the activation of the EGFR-AKT signalling pathway. We then observed the regulatory effects of the SZYQD extract on activation of the EGFR-AKT signalling pathway induced by EGF. The results showed that compared with the blank group, protein expression of p-AKT and p-EGFR was upregulated; in contrast, protein expression of p-AKT and p-EGFR was significantly downregulated after treatment with SZYQD extract (Fig. 5E-F). A previous study demonstrated that activation of EGFR-AKT induced MUC5AC production [25]. Consistently, we found that SZYQD water extract inhibited expression of MUC5AC induced by EGF. Our results show that SZYQD inhibits activation of the AKT signalling pathway and MUC5AC production induced by EGF in airway epithelial cells (Fig. 5G-H).

### The effect of luteolin on IL-6 production and EGFR-AKT pathway activation

**Fig. 6:**
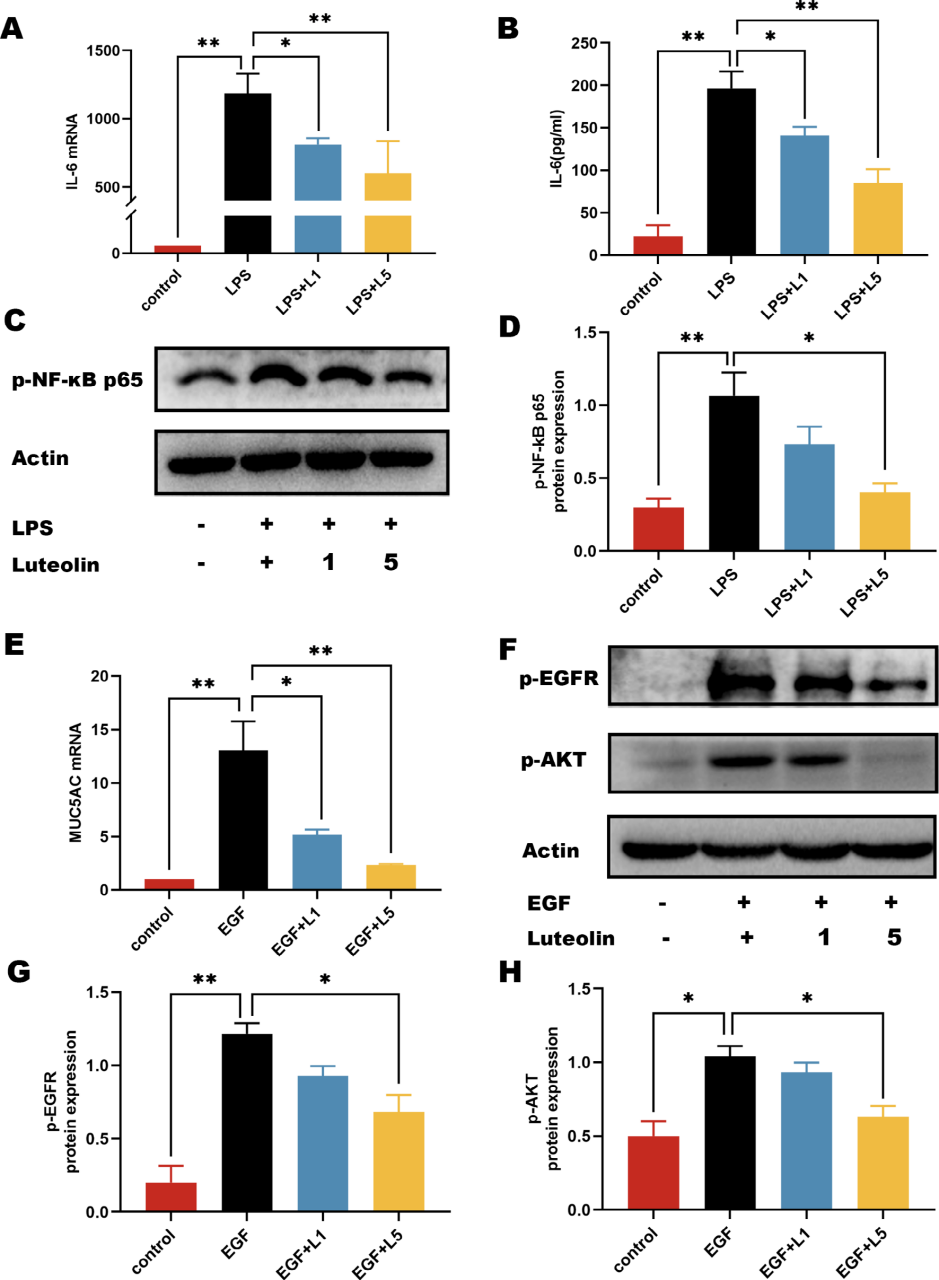
Effects of luteolin on the expressions of IL-6 and AKT activation in M-HS and H292 cells. (**A-B**) M-HS cells were exposed to LPS (1 µg/mL) and luteolin (1 µM and 5 µM) for 24 h. The mRNA expression levels of IL-6 and secretory levels of IL-6 were examined by qPCR and ELISA. (**C**) M-HS cell was exposed to LPS (1 µg/mL) and luteolin (1µM and 5µM) for 1 h. The expression levels of p-p65 were measured by western blot. (**D**) Quantification of p-p65. (**E**) H292 cells were exposed to EGF (50ng/mL) and luteolin (1µM and 5µM) for 24 h. The expression levels of MUC5AC were examined by qPCR. (**F**) The expression levels of p-AKT and p-EGFR protein levels were measured by western blot. (**G-H**) Quantification of p-EGFR and p-AKT. The data were expressed as mean ± SD (n = 3) of each group. *p < 0.05, **p < 0.01

**Fig. 7:**
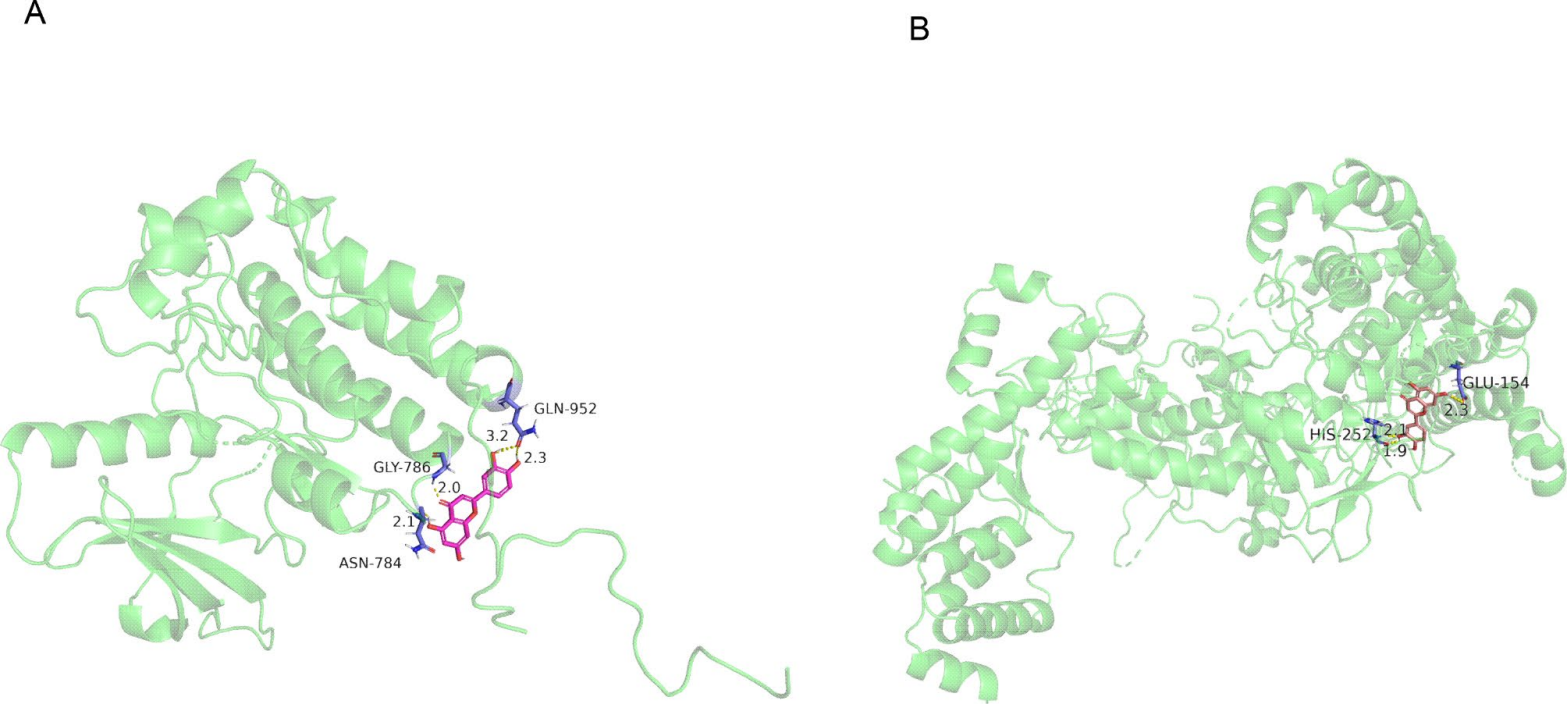
Docking patterns of key targets and specific active compounds. Luteolin-RELA (**A**) and Luteolin-EGFR (**B**)

Active ingredient analysis found that luteolin was the core component of SZYQD (Fig. 2E). The SZYQD water extract was analyzed using UHPLC-QE-MS, and the presence of luteolin was detected (Supplementary Figure S1). We further observed the effect of luteolin on IL-6 expression and EGFR-AKT pathway activation. The results showed that luteolin not only inhibited IL-6 expression and p-p65 induced by LPS (Fig. 6A-D), but also inhibited EGF-induced EGFR-AKT activation and MUC5AC overexpression(Fig. 6E-H). The above results are consistent with the effects of SZYQD water extract. We then used molecular docking technology to evaluate the mutual binding of luteolin and key targets (EGFR, RELA). Compound-target interactions and their binding patterns were visualized using PyMoL. Luteolin is bound by <-3.0 kJ/mol, with low conformational energy, a relatively stable structure, and high binding activity. Through hydrogen bonding, the active cavities of hydrophobic small molecules and target proteins form stable complexes. The docking and binding energies of EGFR and RELA and luteolin were − 3.52 kJ/mol and − 4.69 kJ/mol, respectively, indicating that luteolin could bind to key targets, which might inhibit phosphorylation of target molecules and inhibit pathway activation (Fig. 7). These results showed that luteolin was one of the important active ingredients in SZYQD that inhibited IL-6 production and AKT pathway activation.

## Discussion

SZYQD is a common prescription for the treatment of asthma. However, there are few studies demonstrating the active components and mechanism of SZYQD in the treatment of asthma. In this study, the “SZYQD target-asthma” network was established through network pharmacology, and on the basis of pharmacological analysis, the related effective components and potential mechanism of SZYQD in the treatment of asthma were discussed. Our in vivo and in vitro experiments showed that SZYQD and its main active component luteolin play a therapeutic role in relieving asthma symptoms by reducing inflammation and mucus overproduction, which was associated with decreased IL-6 and MUC5AC expression.

Using OB and DL as the screening conditions, 28 effective active compounds were obtained from the TCMSP database and literature reports, including 5 from Semen raphani, 19 from Perilla frutescens, and 4 from Semen sinapis. It can be preliminarily predicted from the GO enrichment results that the targets of SZYQD for the treatment of asthma mainly involve the biological functions of positive regulation of protein phosphorylation, response to lipopolysaccharide, inflammatory response, asymmetric synapse, cytoplasmic side of membrane, metalloendopeptidase activity, electron transfer activity, and chromatin DNA binding etc. KEGG analysis indicated that SZYQD was predicted to treat asthma by regulating multiple pathways, including Lipid and atherosclerosis signalling pathway, PI3K-Akt signalling pathway, Kaposi sarcoma-associated herpesvirus infection signalling pathway, antiviral infection (hepatitis B) signalling pathway, Human cytomegalovirus infection signalling pathway, AGE-RAGE signalling pathway, Cancer pathway. Core target analysis showed that SZYQD may directly or indirectly affect asthma through potential targets, such as AKT, IL-6, TNF-α, and EGFR (Fig. 3). A recent study demonstrated the role of SZYQD in regulating TH1 and TH2 balance in asthma treatment [26]. They obtained 14 nodes and 61 lines. It was based on the DAVID database, with the species limited to “Homo sapiens”. The results showed IL-4 and TNF-αare the main targets for the treatment of asthma .This article studies based on Metascape database to get a more comprehensive pathway. Previous studies in COPD confirm the role of SZYQD [27].They obtained 104 common targets and screened 6 core targets, including EGFR, MMP9, PTGS2,MMP2, APP, and ERBB2. Consistently, EGFR was found to be the key target of SZYQD in our study. This suggested EGFR as an essential molecular target for SZYQD. Moreover, we also identified IL-6 as an important target of SZYQD, which was associated with the inhibitory response to LPS. These results displayed the diverse roles of SZYQD in asthma treatment.

The increased expression of MUC5AC is an important marker of airway goblet cell proliferation and mucus hypersecretion in asthma [28], and it is also an independent risk factor affecting the development and prognosis of asthma [29]. However, the mechanism by which SZYQD inhibits expression of MUC5AC has not been fully elucidated. Based on network pharmacology analysis, we found that EGFR-AKT is the key target of SZYQD. Meanwhile EGFR-AKT activation had been reported to be closely related to the overexpression of MUC5AC. For example, Xu found reduced MUC5AC expression by inhibiting AKT phosphorylation [30]. Consistently, we found that EGF-induced overexpression of MUC5AC was decreased in the SZYQD group. Furthermore, p-EGFR and p-AKT level were also inhibited after SZYQD treatment. These findings suggest that SZYQD can inhibit AKT phosphorylation and MU5AC protein expression through the EGFR-AKT pathway, which partially explains the mechanism by which SZYQD inhibits MUC5AC expression.

Airway inflammation is a key pathological feature of asthma. Our results, combined with the GO analysis, suggest the involvement of SZYQD in the response to LPS. It is well-known that endotoxin and its purified derivative lipopolysaccharide can exacerbate asthma inflammation. Therefore, we utilized a combined induced asthma mouse model of OVA and LPS to elucidate the mechanistic role of SZYQD in improving asthma. The observed reduction in inflammatory cell infiltration following SZYQD treatment supported its potential as a protective agent against asthmatic inflammation. According to the PPI analysis, inflammatory factor IL-6 was the highest among the related targets of SZYQD in the treatment of asthma. The level of IL-6 in the serum of patients with asthma is elevated; in a study of bronchoalveolar lavage fluid (BALF), IL-6 level in patients with active asthma was higher than that in healthy subjects and patients with stable asthma [31]. Some studies have shown that airway inflammation in asthmatic mice was alleviated after local blockade of the IL-6 receptor signalling pathway with an IL-6Ra antibody and gpl30 fusion protein [32]. For patients with asthma, IL-6 has been found to have a negative correlation with FEV1 [33].and is related to the loss of central airway function [34]. These studies show that IL-6 is not only an inflammatory marker but also closely related to the pathogenesis of asthma. Meanwhile, GO analysis indicated that the function of SZYQD was related to the response to LPS. Endotoxin and its purified derivative LPS are gram-negative bacterial potent pro-inflammatory constituents and have been regarded as enhancing factors for asthma severity. NF-κB activation is closely related to the LPS response [35]. Inhibition of the NF-κB pathway attenuates airway inflammation and IL-6 production in asthmatic mice [36]. Consistently, the disease targets analysis identified RELA (p65, subunit of NF-κB) as targets of SZYQD. We used LPS to induce IL-6 overexpression in M-HS cells. We found that SZYQD extract intervention reduced IL-6 expression in LPS-treated M-HS cells and inhibited the activation of the NF-κB signalling pathway, indicating the anti-inflammatory effect of SZYQD. Data have suggested a link between bacterial infections and the exacerbation of asthma and suggest that bacterial infections may be causative in asthma development [37]. Based on the results, it is likely that SZYQD has a protective effect on the bacterial-induced response, which indicated a new application of SZYQD in the treatment of asthma.

Luteolin is a typical flavonoid that has high oral availability and drug-like properties and is the main source of the core target of SZYQD. Luteolin has anti-inflammatory, anti-allergic, and immune enhancement functions. Some studies have shown that luteolin decreases the expression of IL-4, IL-5 and IL-13 in the bronchoalveolar lavage fluid (BALF) of asthmatic models. It can reduce inflammation and autophagy in lung tissue [38]. Other studies have shown that luteolin can block AKT signal transduction to balance the immune response and reduce inflammatory damage, thus improving the prognosis of asthma and regulating the PI3K/Akt signalling pathway [39, 40]. Our studies showed that luteolin significantly inhibited LPS-induced IL-6 expression and NF-κB pathway activation, EGF-induced MUC5AC expression, and EGF-induced EGFR-AKT signalling pathway activation. Achour M et al. found that luteolin significantly attenuated LPS-induced neuroinflammation by reducing IL-6 production and reducing serum IL-6 in mouse brain-derived astrocytes [41]. Attiq et al. also reported that luteolin abolished the inflammatory response in LPS-treated human plasma, specifically reducing the expression of IL-1 and IL-6 [42]. Choi Eun-Young et al. found that luteolin strongly suppressed the production of NO and IL-6 at both the gene transcription and translation levels in Pi LPS-activated M-HS cells [43]. Our results confirmed the inhibitory effect of luteolin on LPS-induced IL-6 expression and further indicated that the underlying mechanism was associated with NF-κB pathway inhibition. Meanwhile, molecular docking demonstrated that luteolin could bind to p65, which might result in decreased phosphorylation of p65 and NF-κB inhibition. The anti-inflammatory effect of luteolin is well known, but the effect of reducing high mucus secretion is poorly studied. Our experiments further demonstrated that luteolin can effectively reduce the expression of MU5AC, mainly by regulating the EGFR-AKT signalling pathway in airway epithelial cells, thus inhibiting mucin production, therefore secretion.

## Conclusion

Our results show that SZYQD and its key compound luteolin can relieve asthma symptoms through the mechanisms predicted by network pharmacology, such as reducing overexpression of IL-6 and MUC5AC, inhibiting activation of the EGFR-AKT pathway and NF-κB pathway to inhibit inflammation and mucus overproduction. These findings provide a new reference for the clinical application of SZYQD.

### Electronic supplementary material

Below is the link to the electronic supplementary material.

**Supplementary Material 1**: A UHPLC-QE-MS analysis was used for SZYQD water extract to detect the presence of luteolin

**Supplementary Material 2**: Raw data for WB data

**Supplementary Material 3**: Data for Table

## Data Availability

The datasets used and/or analyzed during the current study are available from the corresponding author upon reasonable request. Data in this paper are available using publicly available databases: TCMSP database (http://tcmspw.com/tcmsp.php).UniProt database (https://www.uniprot.org/).Gene Cards database (http://www.genecards.org/) and OMIM database (https://omim.org/). KEGG database (http://www.genome.ad.jp/kegg/).
