# Supplementary figures and images for: The mechanism of Sanzi Yangqin decoction for asthma treatment based on network pharmacology and experimental verification
Source: BMC Complement Med Ther. 2023 Dec 13;23:452. doi: 10.1186/s12906-023-04272-6 (PMC10717567; doi:10.1186/s12906-023-04272-6)

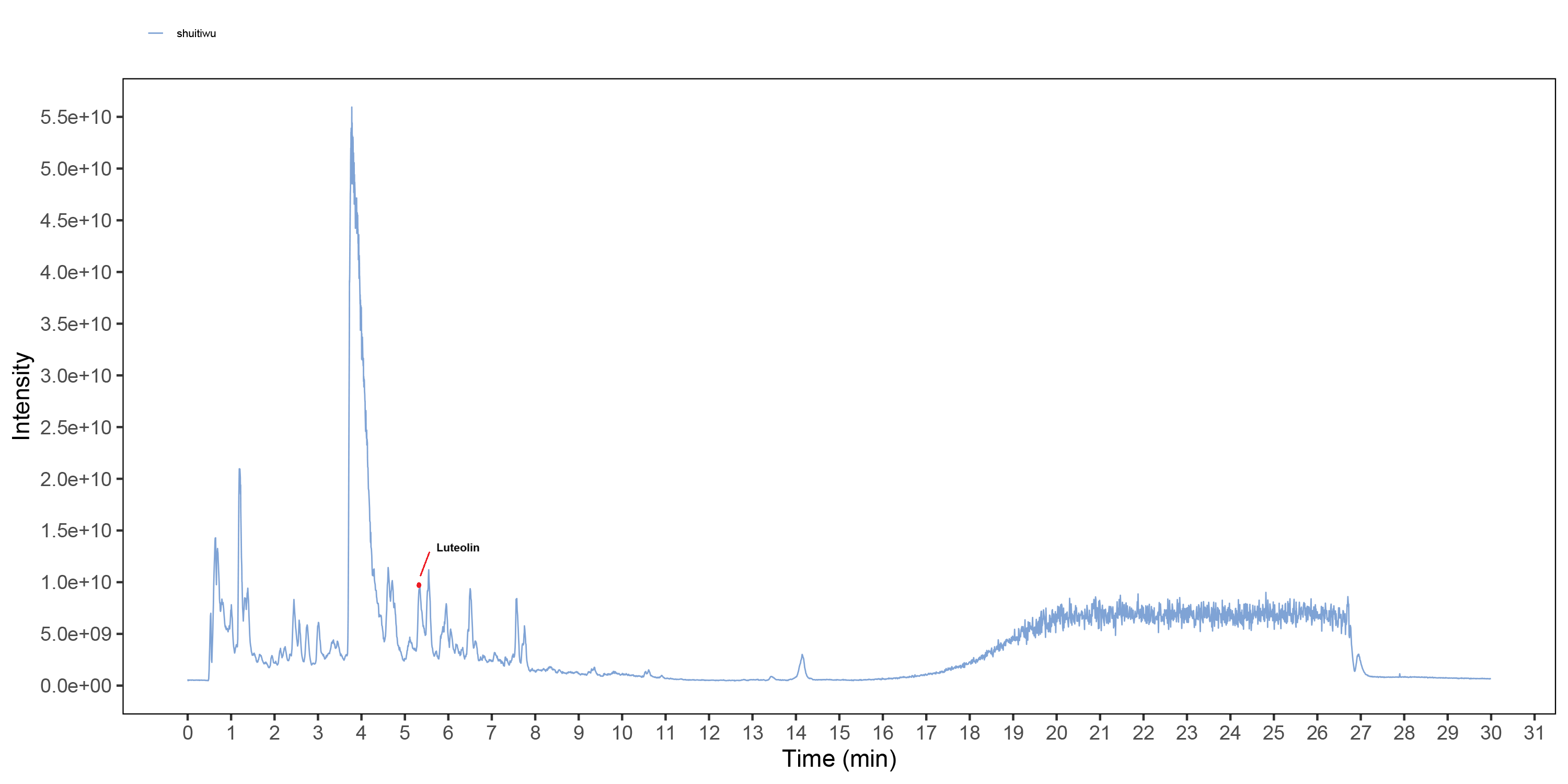

Supplement: Supplementary file 1 — Supplementary Material 1: A UHPLC-QE-MS analysis was used for SZYQD water extract to detect the presence of luteolin [file 12906_2023_4272_MOESM1_ESM.tif]

Fig 4 F (mice)

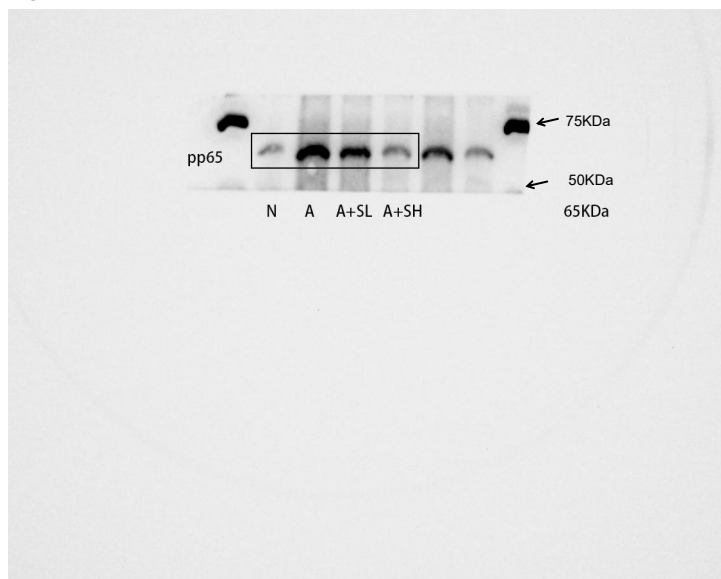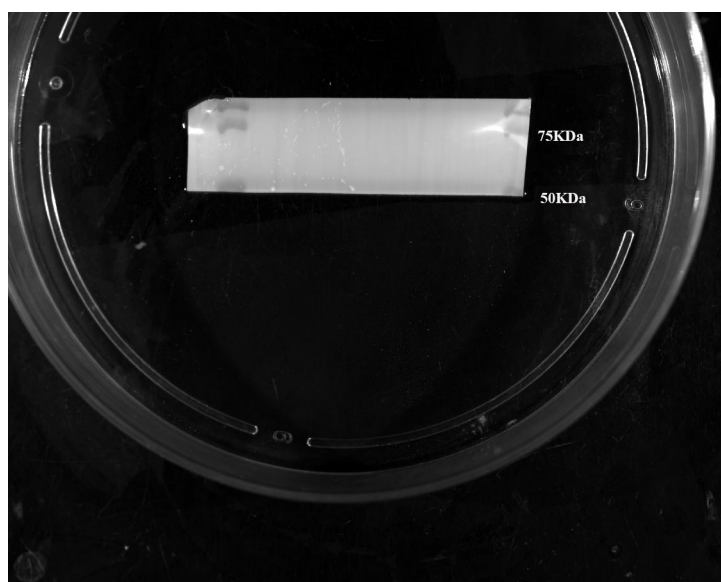

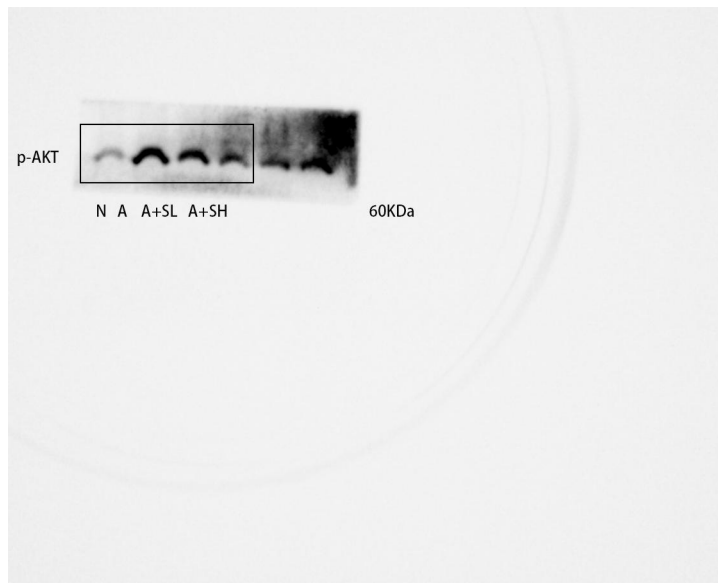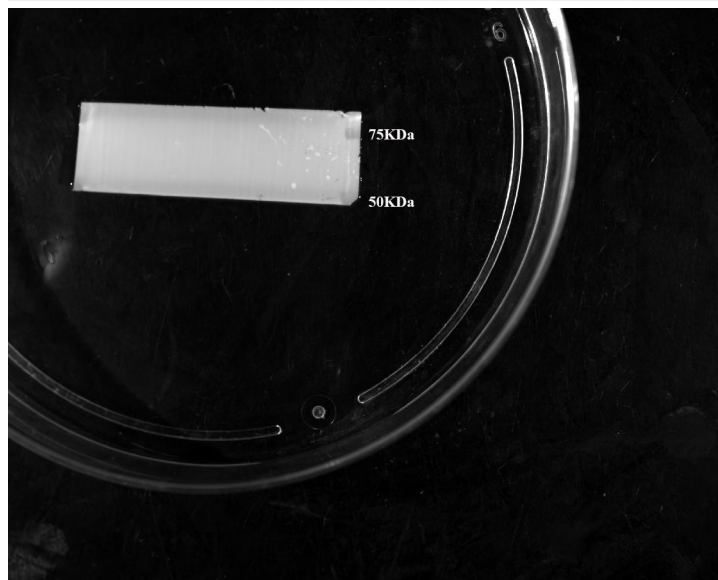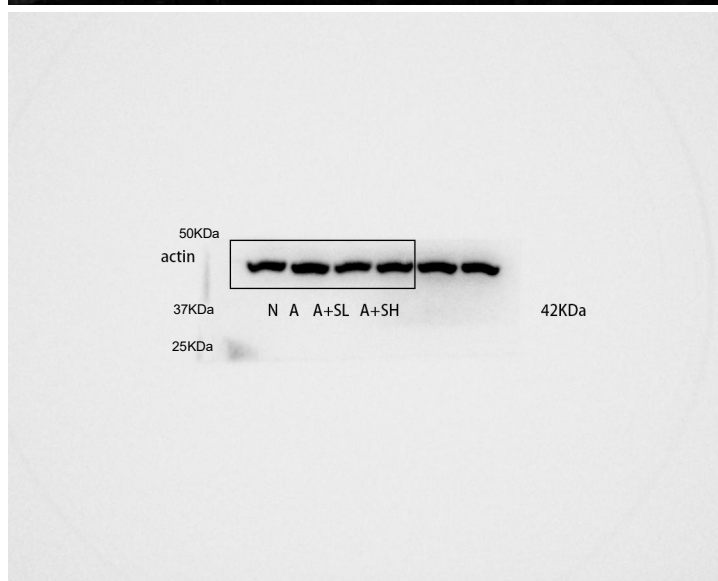

Fig 5C (cell)

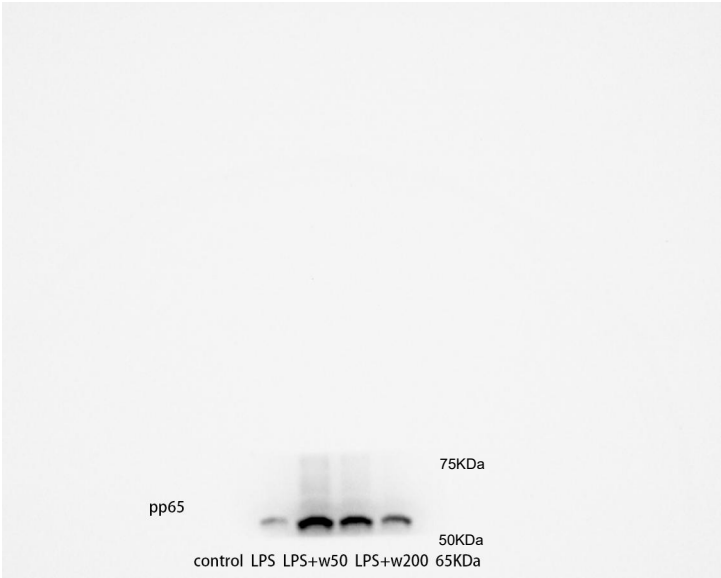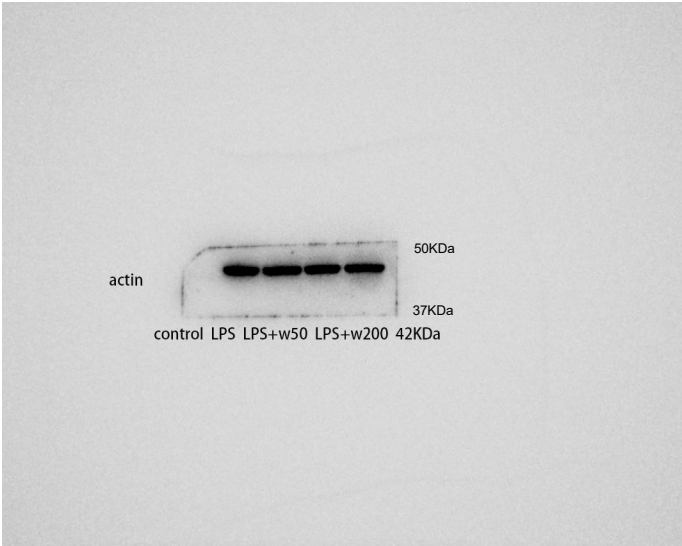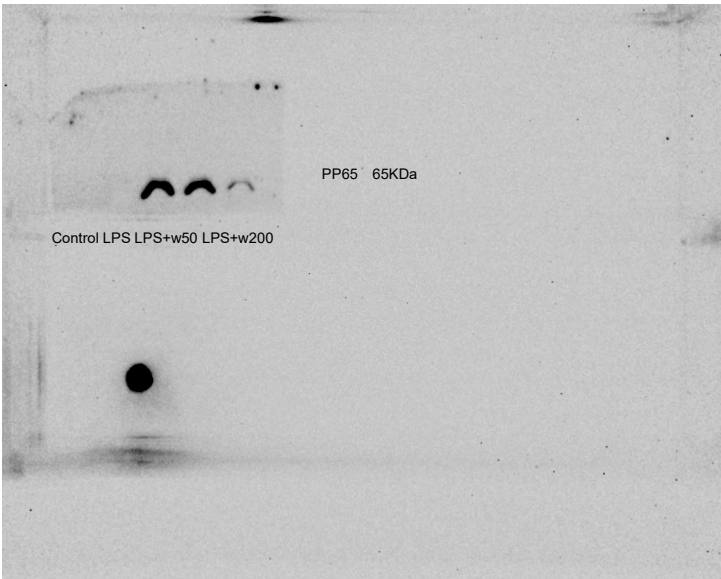

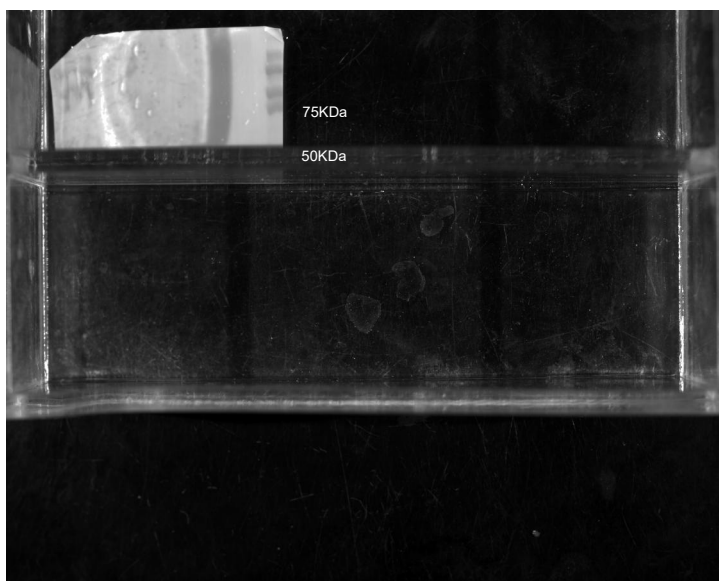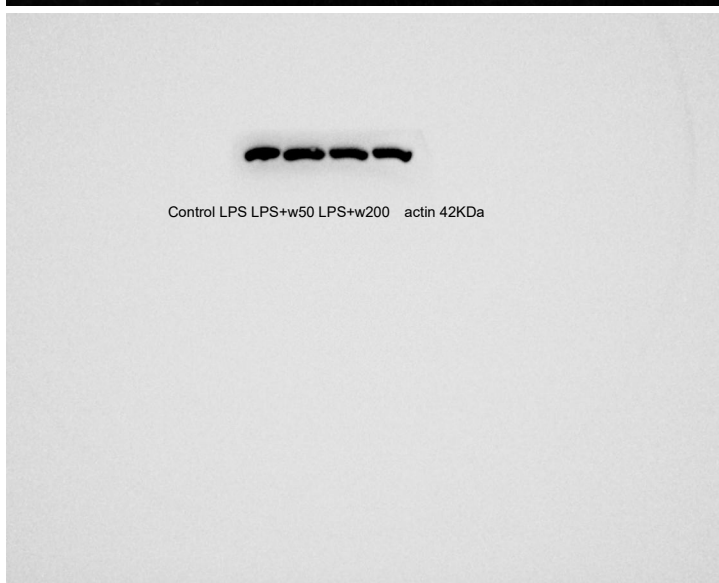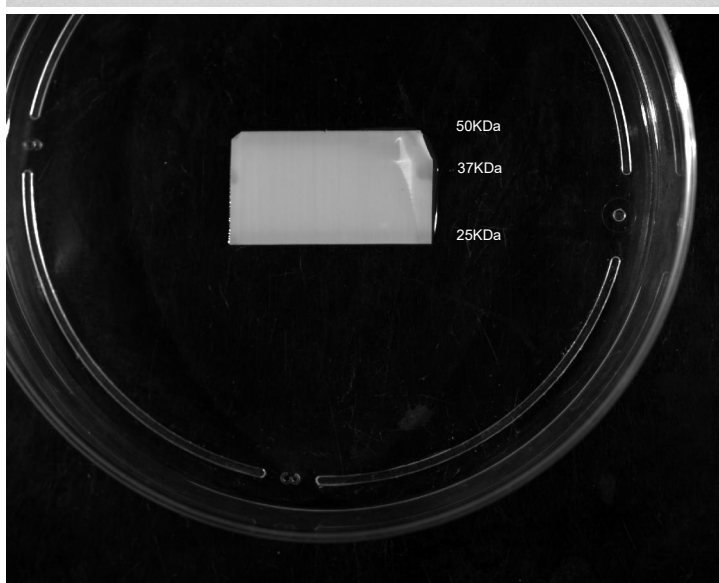

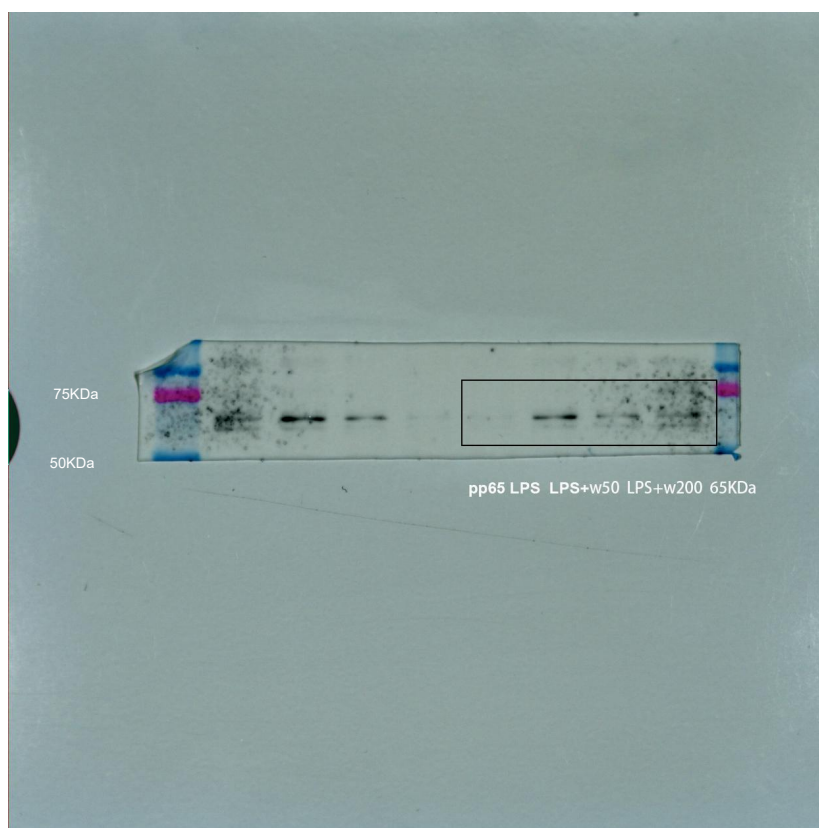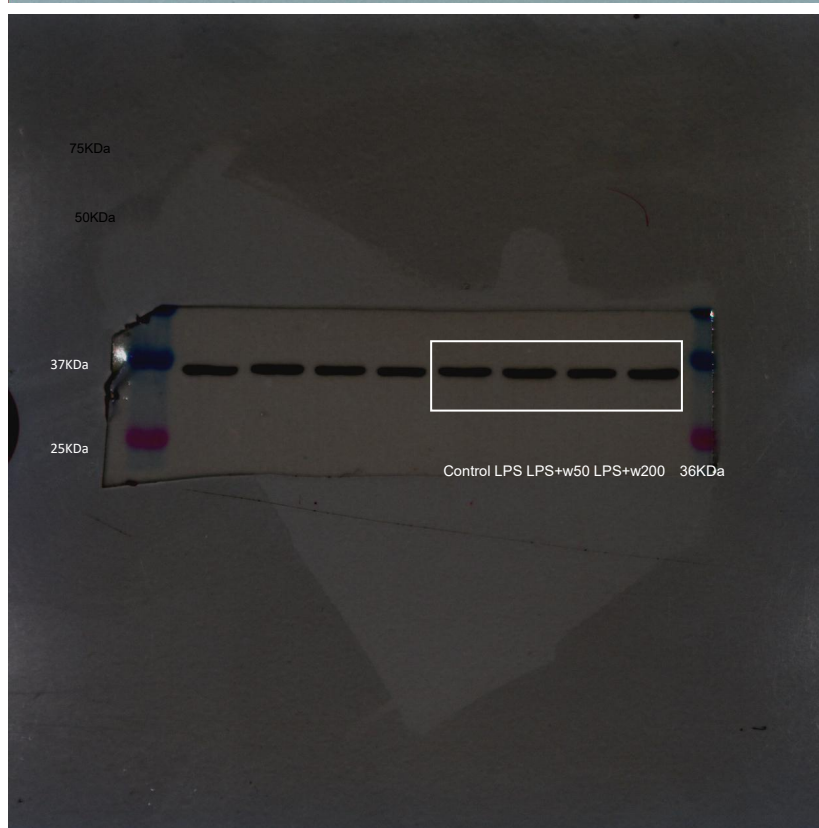

Fig 5F

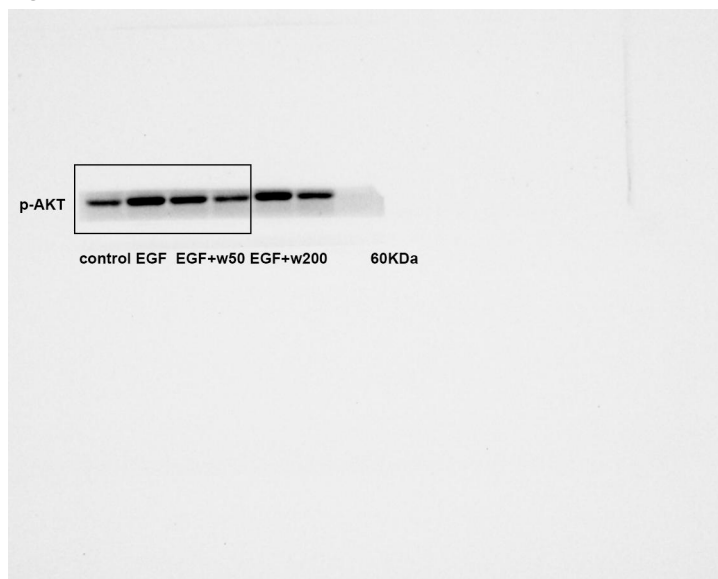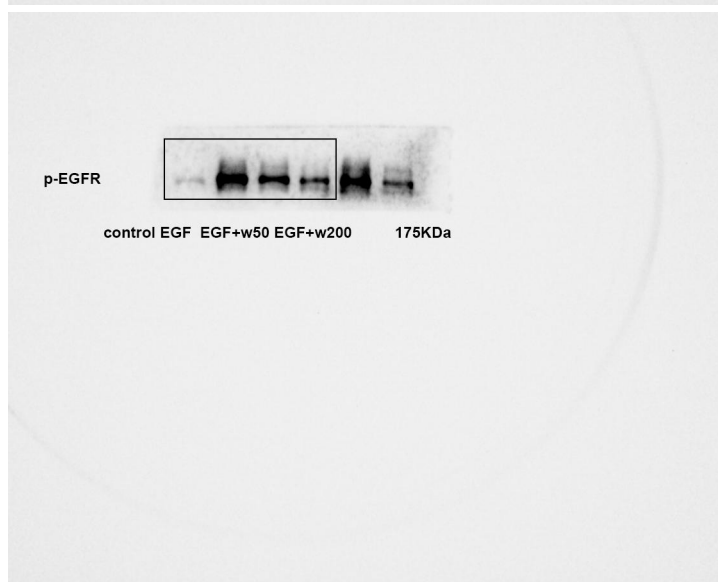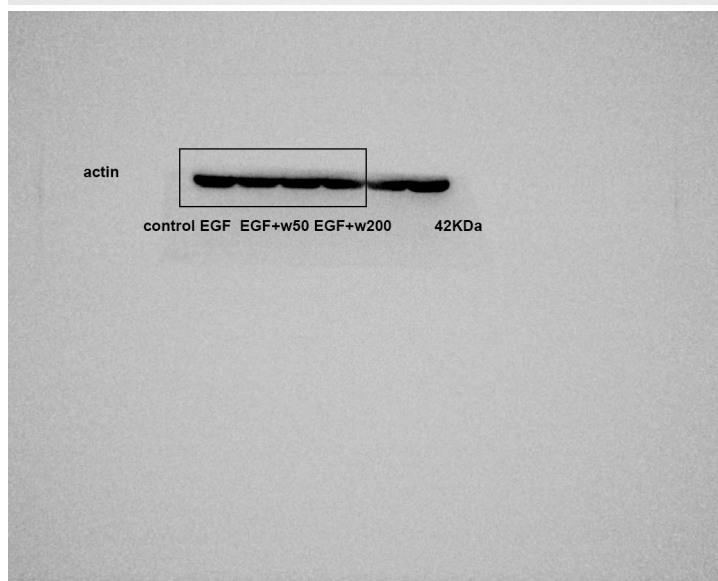

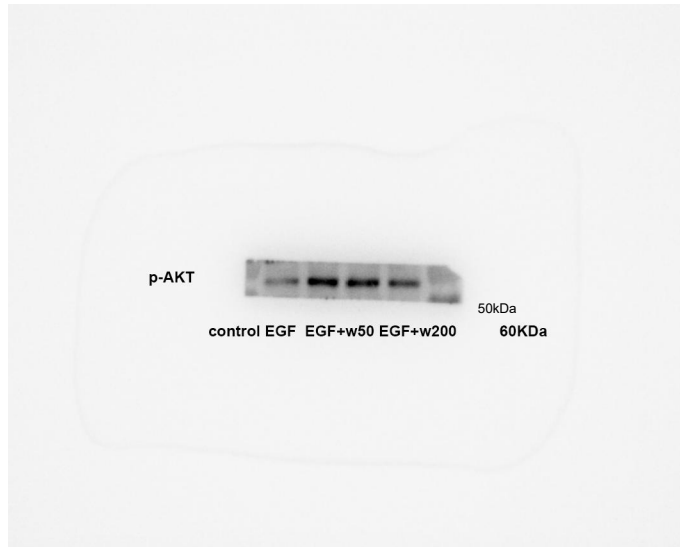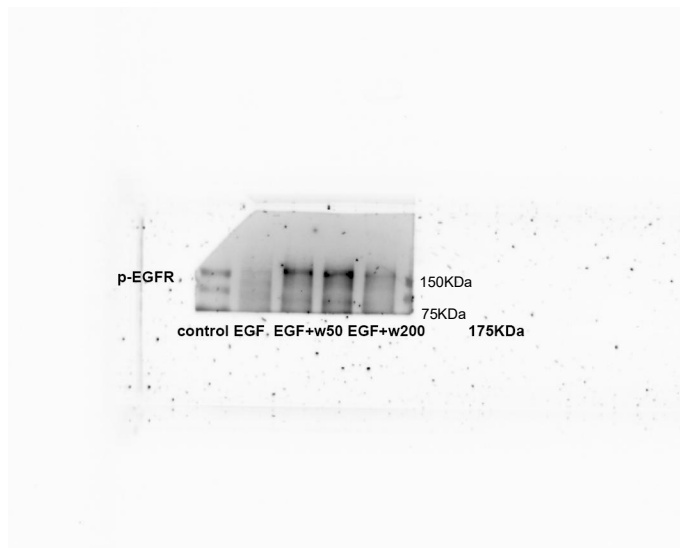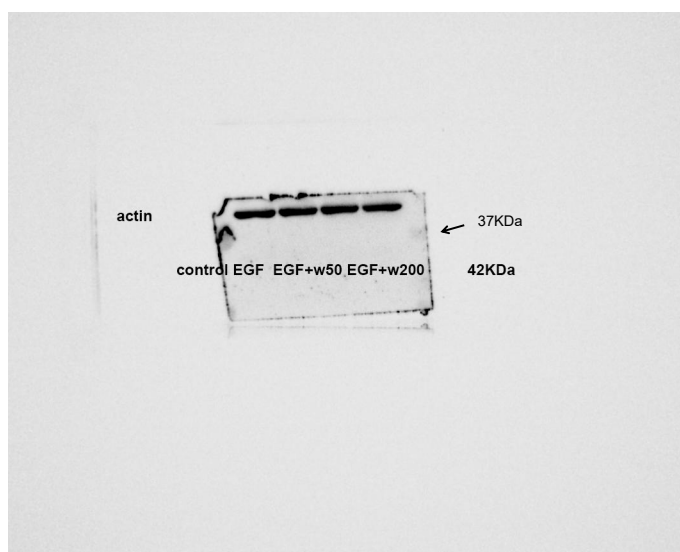

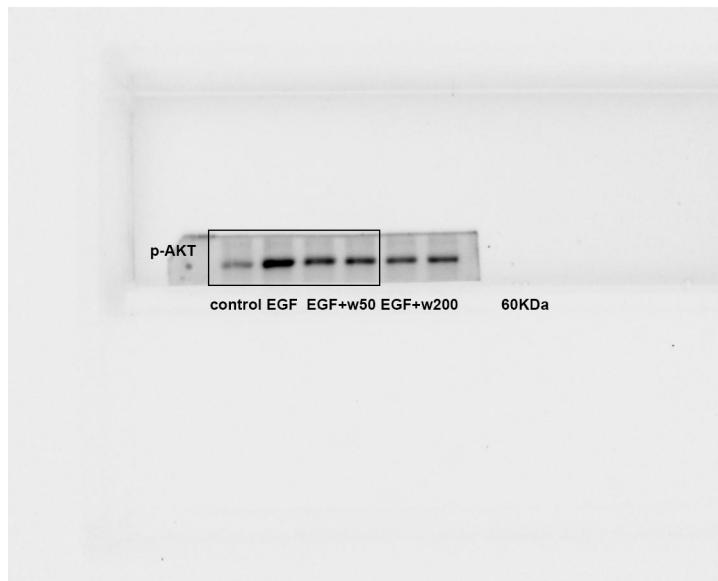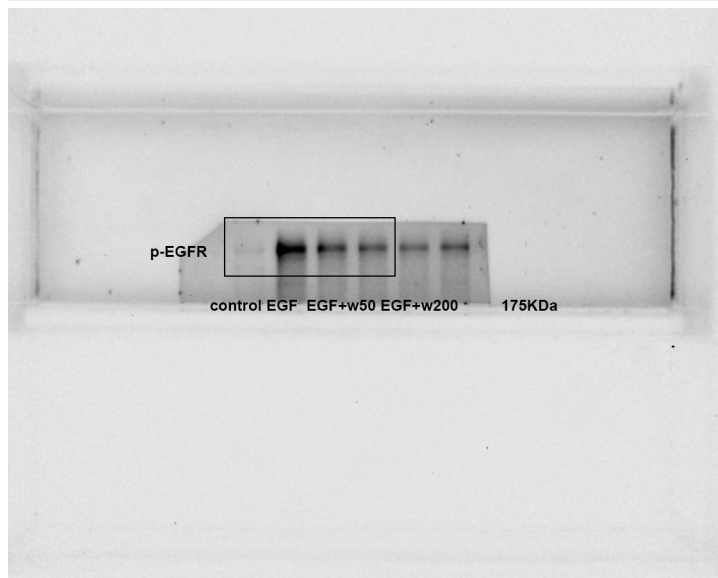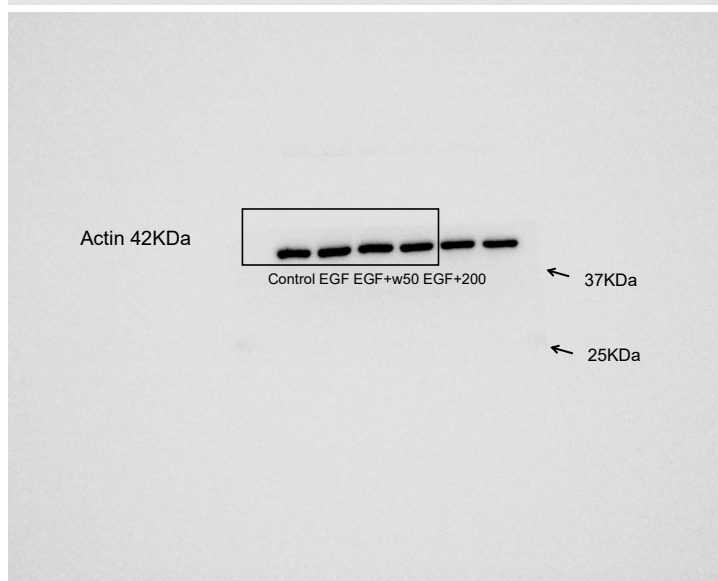

Fig 7C

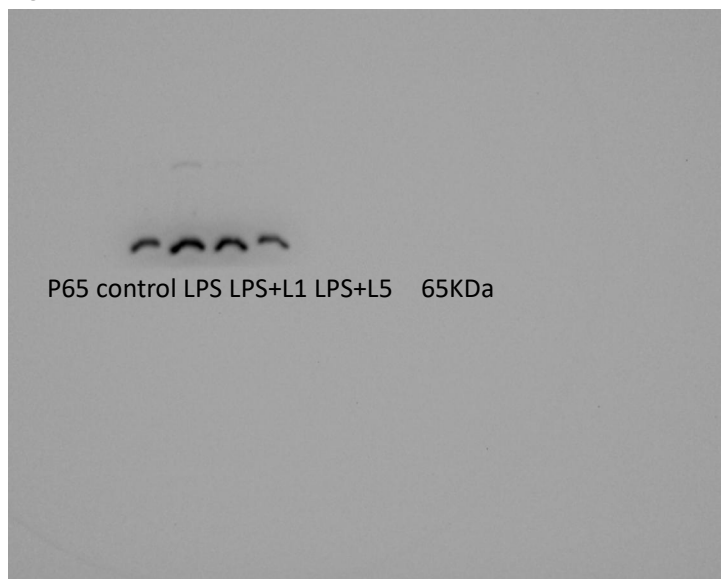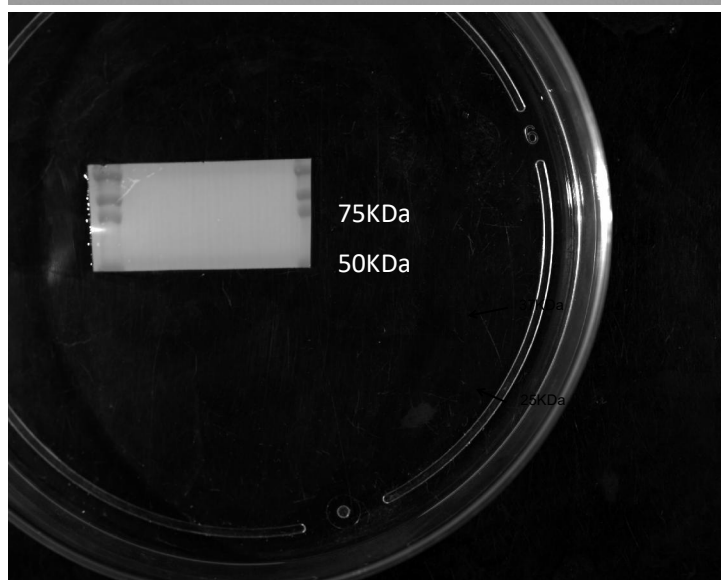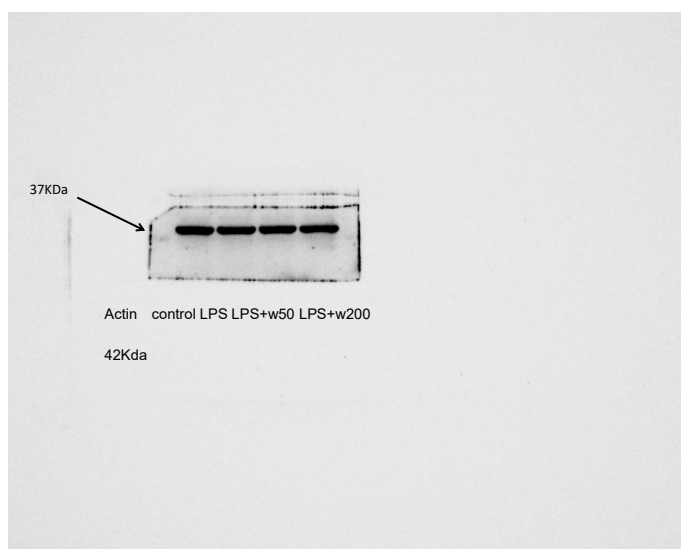

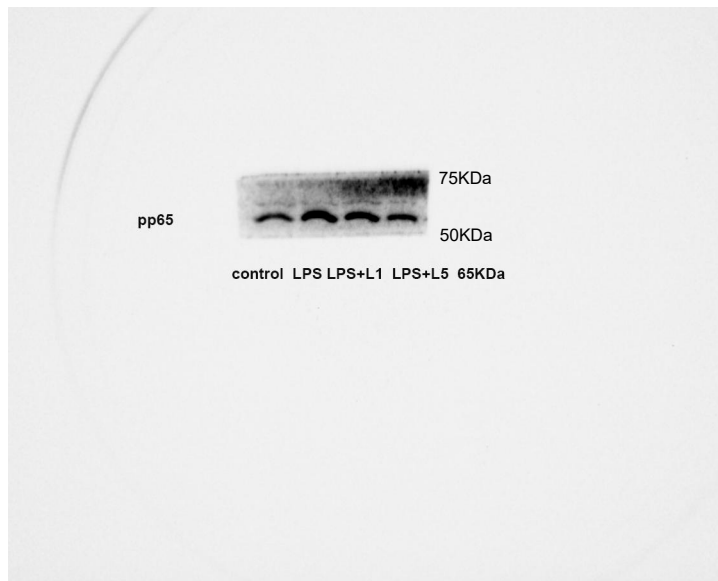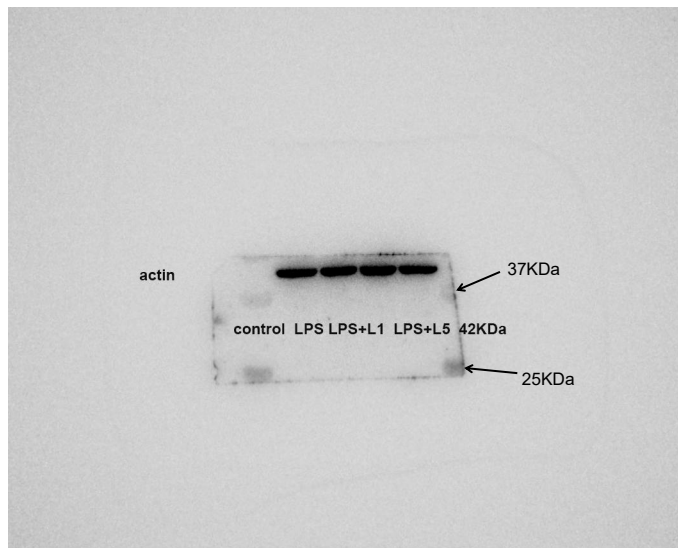

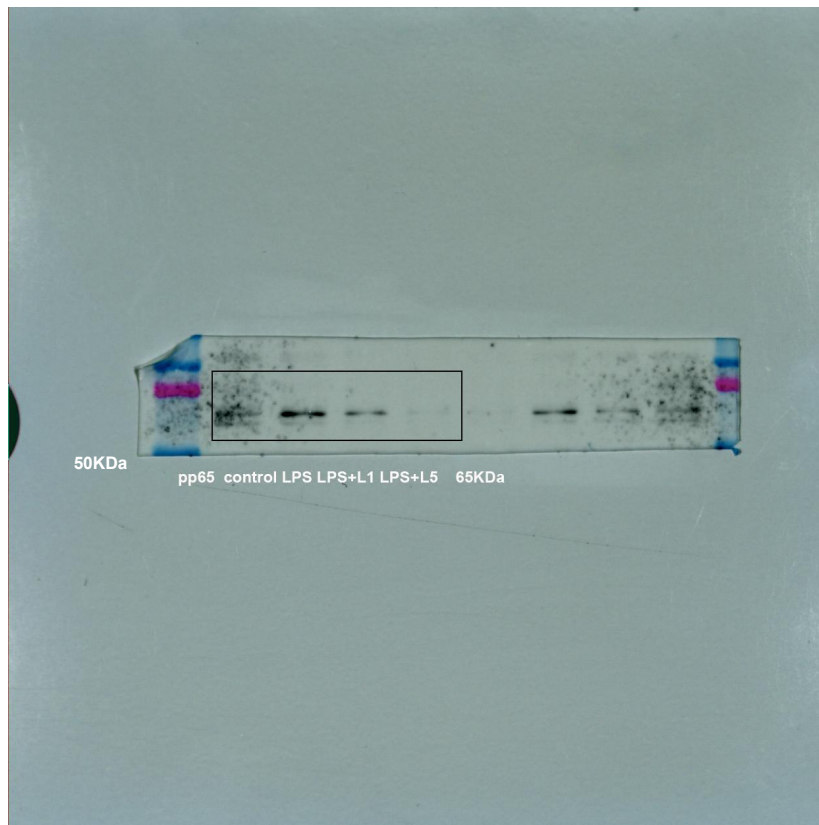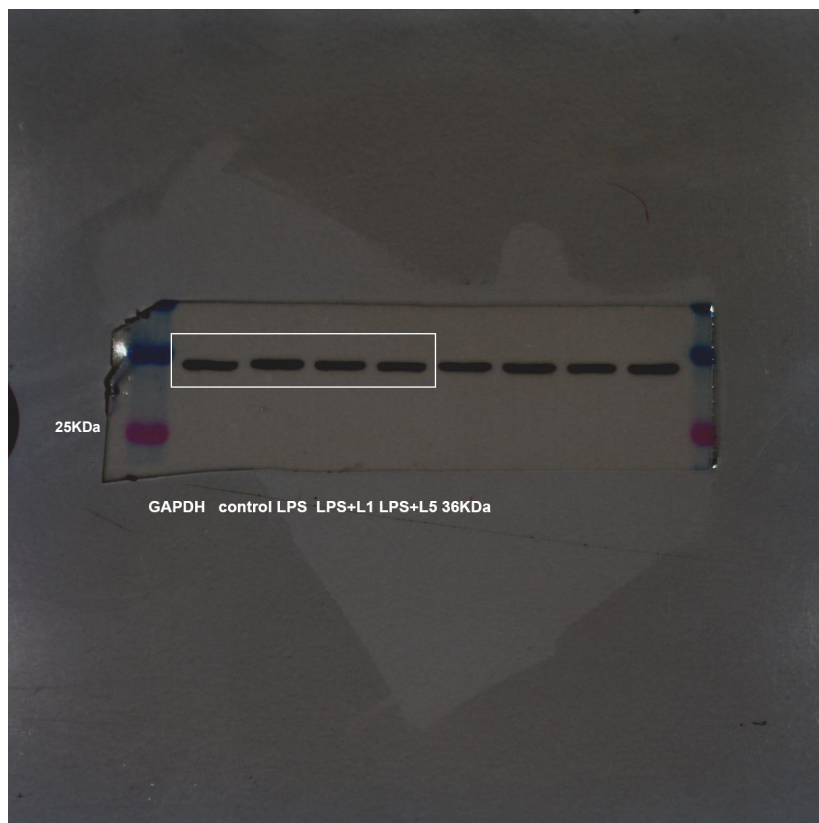

Fig 7F

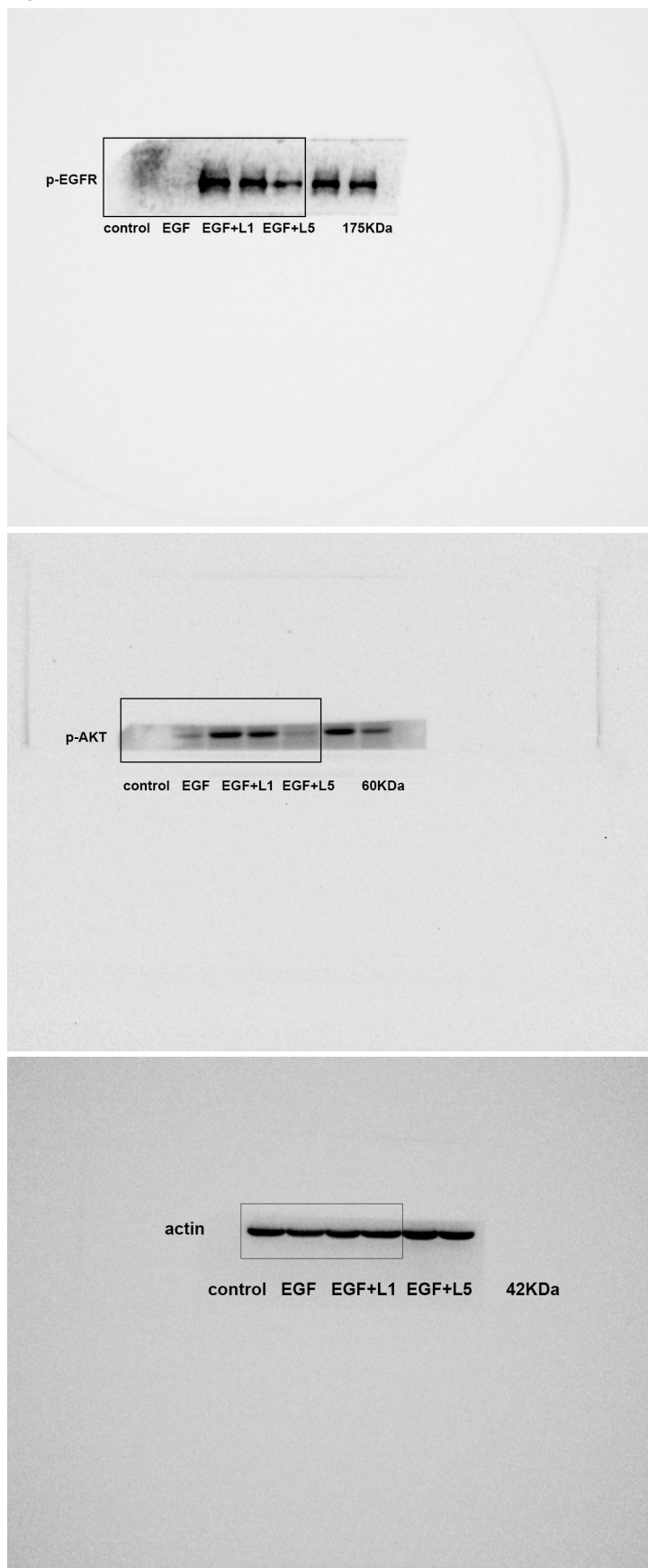

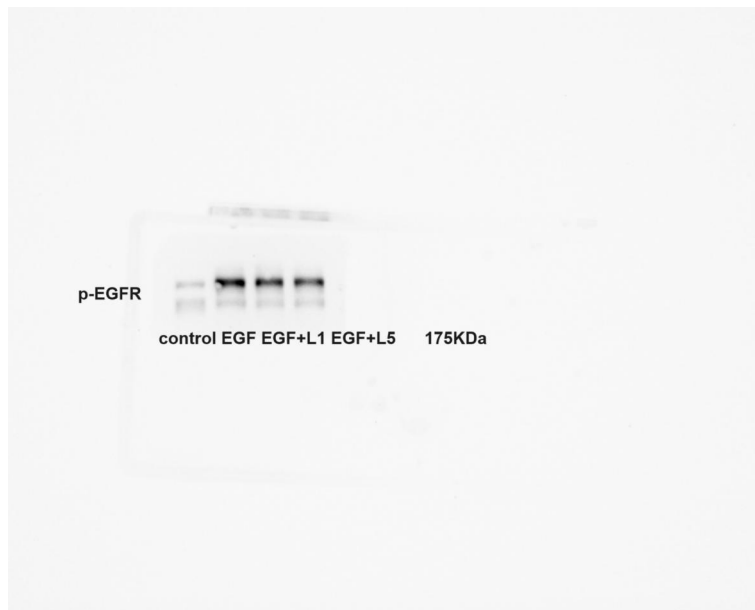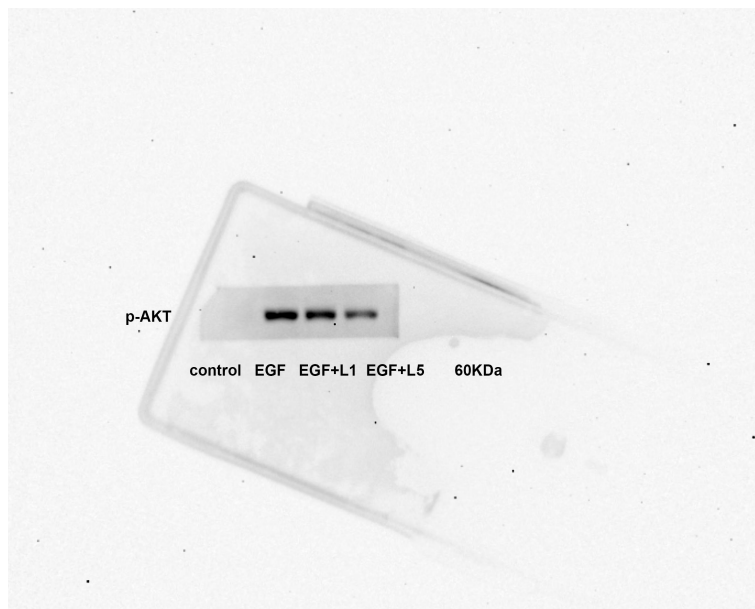

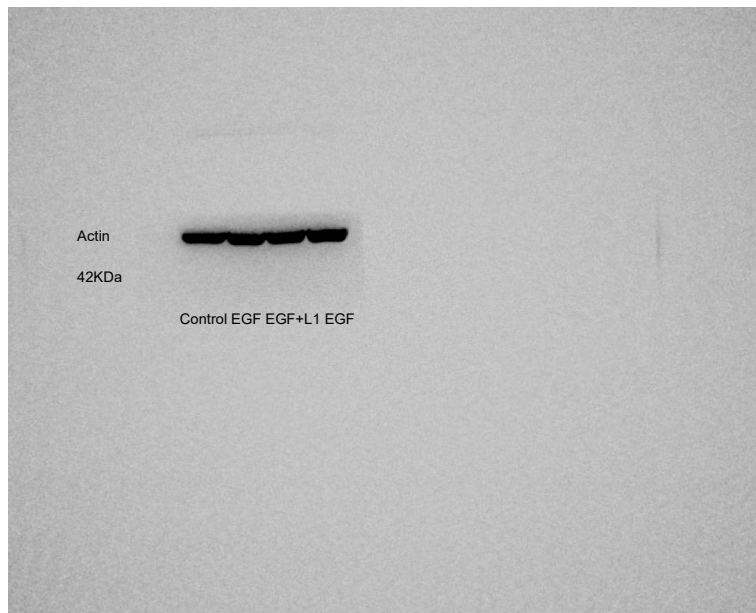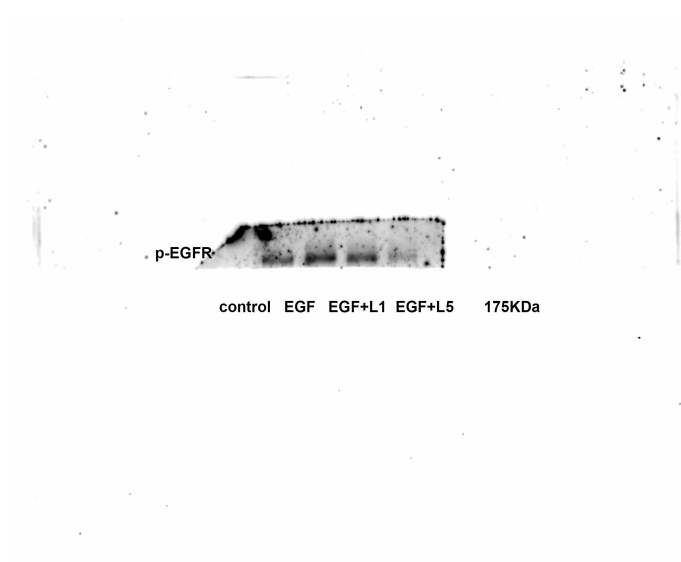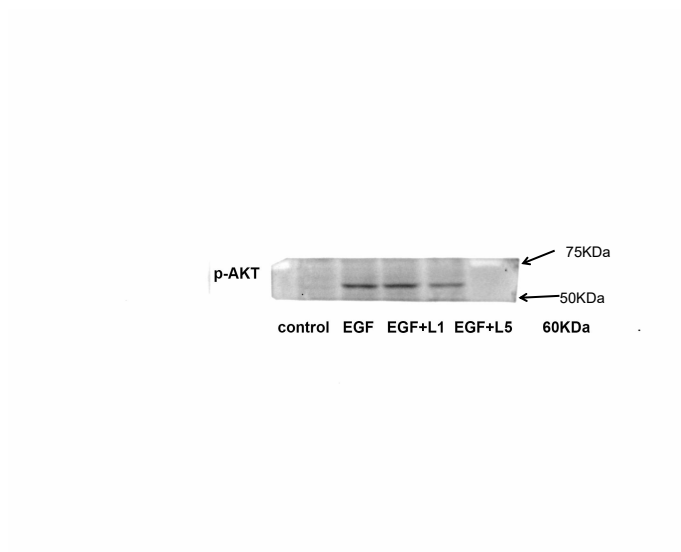

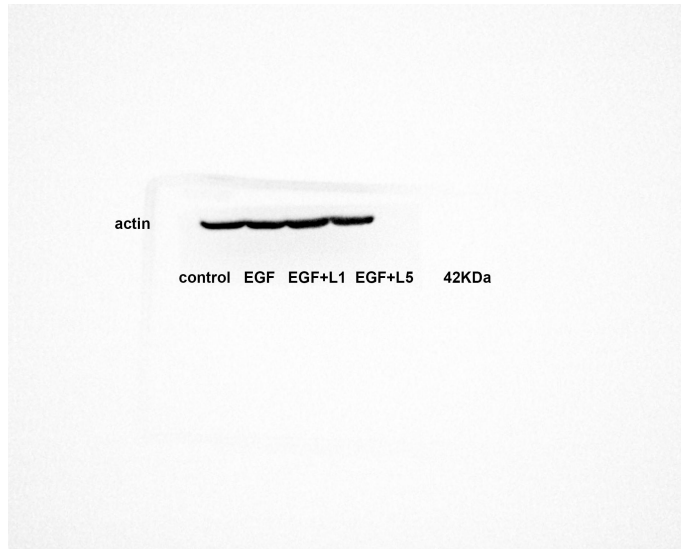

Supplement: Supplementary file 3 — Supplementary Material 3: Data for Table [file 12906_2023_4272_MOESM3_ESM.pdf]
