# Supplementary material for: The mechanism of Sanzi Yangqin decoction for asthma treatment based on network pharmacology and experimental verification
Source: BMC Complement Med Ther. 2023 Dec 13;23:452. doi: 10.1186/s12906-023-04272-6 (PMC10717567; doi:10.1186/s12906-023-04272-6)
Supplement: Supplementary file 2 — Supplementary Material 2: Raw data for WB data [file 12906_2023_4272_MOESM2_ESM.doc]

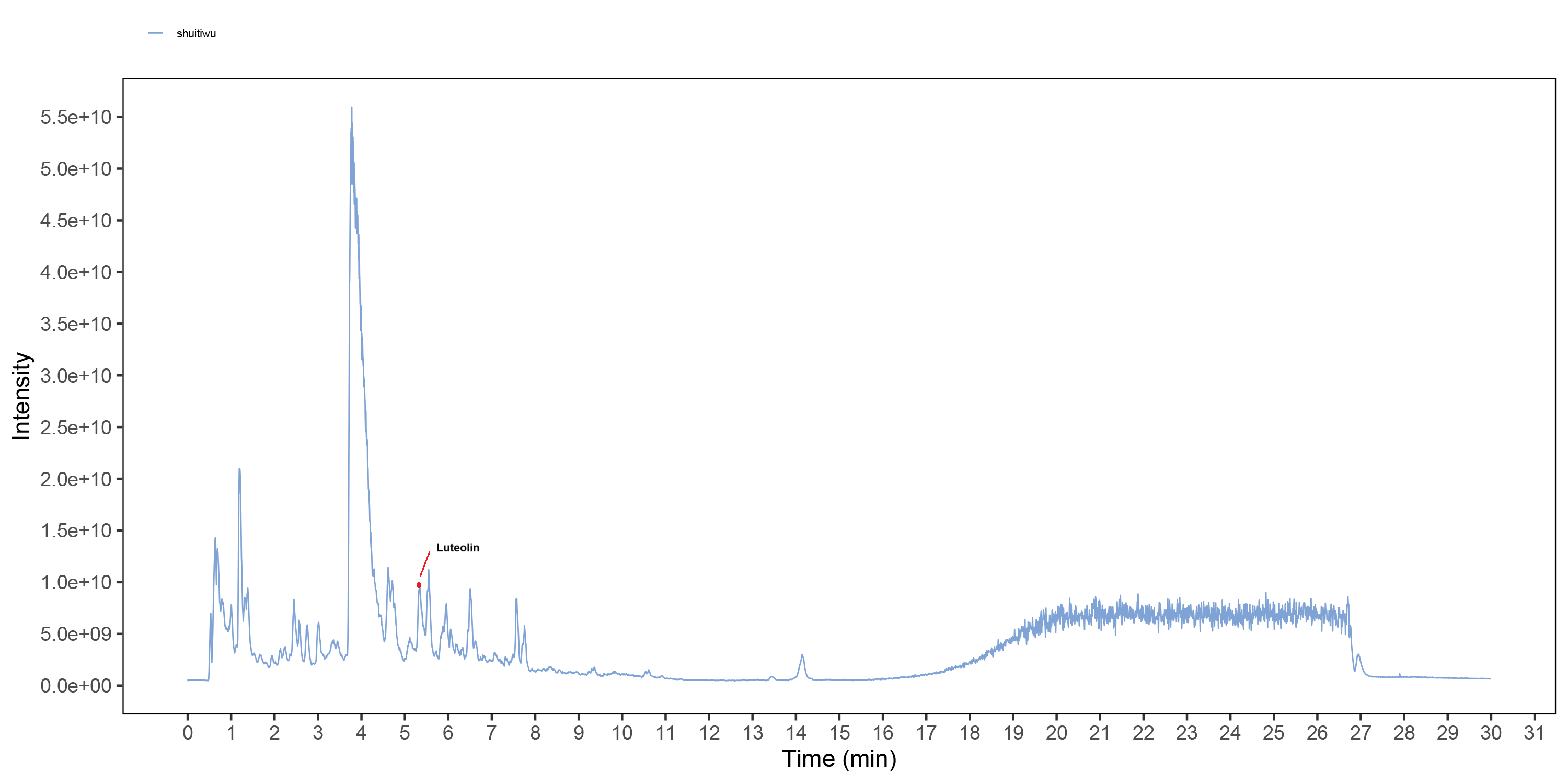


Supplementary Figure S1 legend

The composition of SZYQD extract was identified by UHPLC-QE-MS, the red mark indicates the peak of luteolin.
